# Genome-Wide Gene Expression Profile Analyses Identify CTTN as a Potential Prognostic Marker in Esophageal Cancer

**DOI:** 10.1371/journal.pone.0088918

**Published:** 2014-02-14

**Authors:** Pei Lu, Junjing Qiao, Wei He, Jin Wang, Yongxu Jia, Yan Sun, Senwei Tang, Li Fu, Yanru Qin

**Affiliations:** 1 Department of Clinical Oncology, the First Affiliated Hospital, Zhengzhou University, Zhengzhou, China; 2 Department of Medicine and Therapeutics, the Chinese University of Hong Kong, Hong Kong, China; University of Texas MD Anderson Cancer Center, United States of America

## Abstract

**Aim:**

Esophageal squamous cell carcinoma (ESCC) is one of the most common fatal malignances of the digestive tract. Its prognosis is poor mainly due to the lack of reliable markers for early detection and prognostic prediction. Here we aim to identify the molecules involved in ESCC carcinogenesis and those as potential markers for prognosis and as new molecular therapeutic targets.

**Methods:**

We performed genome-wide gene expression profile analyses of 10 primary ESCCs and their adjacent normal tissues by cDNA microarrays representing 47,000 transcripts and variants. Candidate genes were then validated by semi quantitative reverse transcription-PCR (RT-PCR), tissue microarrays (TMAs) and immunohistochemistry (IHC) staining.

**Results:**

Using an arbitrary cutoff line of signal log ratio of ≥1.5 or ≤−1.5, we observed 549 up-regulated genes and 766 down-regulated genes in ESCCs compared with normal esophageal tissues. The functions of 302 differentially expressed genes were associated with cell metabolism, cell adhesion and immune response. Several candidate deregulated genes including four overexpressed (CTTN, DMRT2, MCM10 and SCYA26) and two underexpressed (HMGCS2 and SORBS2) were subsequently verified, which can be served as biomarkers for ESCC. Moreover, overexpression of cortactin (CTTN) was observed in 126/198 (63.6%) of ESCC cases and was significantly associated with lymph node metastasis (P = 0.000), pathologic stage (P = 0.000) and poor survival (P<0.001) of ESCC patients. Furthermore, a significant correlation between CTTN overexpression and shorter disease-specific survival rate was found in different subgroups of ESCC patient stratified by the pathologic stage (P<0.05).

**Conclusion:**

Our data provide valuable information for establishing molecules as candidates for prognostic and/or as therapeutic targets.

## Introduction

Esophageal squamous cell carcinoma (ESCC) is the predominant type of esophageal cancer (EC) internationally, accounting for more than 90% of all EC cases [1]. ESCC is one of the most common forms of cancer and is associated with a poor prognosis [2]. Despite advances in diagnostic techniques and multimodal therapies, ESCC remains a deadly cancer with a 5-year survival rates averaging 15% in many countries [3]. The bad prognosis is the result of the aggressive biological character of the tumor, a lack of reliable diagnostic techniques for early-stage detection and absence of effective individualized treatment [4]. Decreasing the mortality rate would require early diagnosis and treatment for patients. However, current biomarkers that are used to identify ESCC patients at an early stage and select treatment modalities for individual patients still lack sufficient sensitivity and specificity [5]. So far, molecular technique has been regarded as an ideal method for early diagnosis and prognostic prediction in ESCC. Thus, it is of great clinical value to look for new diagnostic, therapeutic indicators and prognostic prediction based on the molecular mechanism underlying esophageal carcinogenesis.

To understand the molecular basis and select candidates for the development of novel anti-tumor drugs and tumor markers, it is necessary to analyze the global changes of gene expression between the ESCC tumor tissues and non-tumor tissues. For this purpose, cDNA microarray technology, a powerful tool for large scale gene expression studies which based on hybridization, was used to analyze the changes in the expression of thousands of genes simultaneously [6]. Several microarray studies have investigated the gene expression profiles in ESCC tissues and cell lines [7], [8]. However, useful information derived from those studies was limited.

With an aim to identify more novel esophageal cancer-related genes which might be potential molecular targets for diagnosis, treatment, and/or prevention of ESCC, we compared gene expression profiles between tumor tissues and matched normal epithelia from 10 ESCC specimens using the Affymetrix human genome U133 Plus 2.0 GeneChip consisting of 47,000 transcripts. The present study identified a number of differentially expressed genes including those related to cell adhesion, cell metabolism and immune response. Notably, an actin filament-binding protein and a kinase target, cortactin (CTTN or EMS1), was found to be one of the most significant overexpressed genes in ESCC. Here, we reported that overexpression of CTTN has independent prognostic value and could also be served as a therapeutic target for ESCC.

## Materials and Methods

### Ethics Statement

ESCC tissue specimens used in this study were approved by the Committees for Ethical Review of Research involving Human Subjects at Zhengzhou University. Written informed consents for the original human work that produced the tissue samples were obtained.

### ESCC Clinical Samples

Primary ESCC tissues and adjacent normal esophageal epithelial tissues (taken 5 cm away from the tumor edge) were collected at the time of surgical resection at Linzhou People’s Hospital (Henan, China). All tumor tissues were histopathologically confirmed as ESCC. The patients were all previously untreated (i.e, no radiotherapy or chemotherapy). Informed consent was obtained from all patients. The specimens after surgical resection were stored at −80°C immediately until the time of RNA extraction. The ten tumor samples selected for cDNA microarray analyses were derived from dissected tumor tissues which comprised more than 80% of tumor cells without necrosis. Paired adjacent normal mucosas were used as reference. A total of 231 formalin-fixed and paraffin-embedded ESCCs and their corresponding normal esophageal epithelial tissue samples were also kindly provided by Linzhou People’s Hospital. All clinical information was obtained from medical records.

### Cell Lines

Five Japanese ESCC cell lines (KYSE140, KYSE410, KYSE180, KYSE30 and KYSE510) were obtained from DSMZ (Braunschweig, Germany), the German Resource Centre for Biological Material [9]. Chinese ESCC cell line HKESC1,EC18 and EC109 were kindly provided by Professor Srivastava (Department of Pathology, The University of Hong Kong, Hong Kong, China) [10]. All these ESCC cell lines were cultured in RPMI 1640 supplement with 10% fetal bovine serum. The cells were incubated at 37°C in a humidified chamber containing 5% CO_2_.

### RNA Extraction, Amplification and Labeling

Tissue samples were ground into powder in liquid nitrogen, and TRIzol reagent was added as soon as nitrogen vaporization concluded. Total RNA was subsequently isolated according to the manufacturer’s instructions. RNA concentration was measured using a NanoDrop spectrophotometer (ND-1000; Labtech International, France) and the purity was assessed by the A260/A280 and A230/A260 ratios. An Agilent bioanalyzer (model 2100; Agilent Technologies, Santa Clara, CA) was used to assess RNA quality after isolation and, subsequently, after biotin labeling and fragmentation. For this, 100 ng RNA of each specimen was amplified to obtain cRNA and was labeled using Affymetrix GeneChip eukaryotic 2 cycle labeling assays for expression analysis(Affymetrix;900494) according to the manufacturer’s instructions at http://www.affymetrix.com/products/reagents/specific/cdna2.affx.

### Microarray Hybridization

Affymetrix HG U133 Plus 2.0 oligonucleotide microarrays were prehybridized in hybridization solution containing 1 mol/L NaCl, 20 mmol/L EDTA, 100 mmol/L 2-(N-morpholino) ethanesulfonic acid, and 0.01% Tween 20 for 10 minutes at 45°C and 60 revolutions per minute. The prehybridization solution was then removed and replaced with 200 mL hybridization solution containing 0.05 mg/mL fragmented cRNA, and the arrays were hybridized for 16 hours at 45°C and 60 revolutions per minute. Arrays were subsequently washed (Affymetrix fluidics station model 400), and was scanned and visualized using a Gene Array scanner (Hewlett-Packard).

### Microarray Analysis

The quality of the pooled normal or tumor samples have been checked by the heatmap with clustering (Figure S1). Affymetrix human genome U133 Plus 2.0 GeneChip (Affymetrix), covering 47,000 transcripts and variants, was used to identify differentially expressed genes between ESCC tumor tissues and normal tissues. Microarray reaction was done according to the manufacturer’s instructions. Probe set intensities were calculated using the Microarray Analysis Suite (MAS v5.0, Affymetrix) software and normalized against 100 housekeeping genes to a mean intensity of 2,000 in mask files of U133 Plus 2.0 before further statistical analysis. Normalized expression values were then compared between ESCC samples and their paired normal tissues. Fold-change differences were calculated to identify up-regulated and down-regulated genes. Transcripts with more than a 1.5-fold difference in expression level were defined as differentially expressed. Specifically designed online tools, including FatiGO [11], Gene Ontology [12] provided by the GO Consortium, and NetAffx Analysis Center database [13], were used to classify the functional roles of the identified differentially expressed genes. cDNA microarray data have been submitted to Gene Expression Omnibus (GEO, http://www.ncbi.nlm.nih.gov/geo/) under the accession number GSE33810.

### Semiquantitative Reverse Transcription-PCR (RT-PCR)

To examine the reliability of the microarray data, RT-PCR analysis was used to confirm the microarray analysis data for the selected four up-regulated genes(CTTN, DMRT2, MCM10 and SCYA26) and two down-regulated genes (HMGCS2 and SORBS2) as described previously [14]. A total of 2 µg mRNA aliquot from each sample were reverse transcribed to single-stranded cDNAs using an Advantage RT for PCR kit (Clontech) and cDNA was subjected to PCR for 30 cycles of amplification. RT-PCR experiments were carried out with the following sets of synthesized primers specific to the selected six genes or with GAPDH-specific primers as an internal control: cortactin (CTTN or EMS1)-Fr: 5′-TGAGTGTGT GTTCTTCCCCAAG-3′,cortactin(CTTN or EMS1)-Rr:5′-CACGTGACCTTCTGGA AAGACA-3′;DMRT-Fr:5′-GCGTGGTGTCCTGCCTGAAG-3′,DMRT-Rr:5′-GCCCCTTCTTGTCCTCGGTG-3′;MCM10-Fr:5′-GCAAAAATCCCCTGTAGAGA-3′, MCM10-Rr:5′-CCCCACAATTTGACCTCTAG-3′;SCYA26-Fr:5′-CACCTTGGAACTGCCACACG-3′,SCYA26-Rr:5′-TGGGTACAGACTTTCTTGCC-3′;HMGCS2-Fr:5′-CTGGGATGGTCGTTATGCCA-3′,HMGCS2-Rr:5′-TCGTCAAGGGTGAAGGGTCG-3′;SORBS2-Fr:5′-ACGTAGAGAAACTCACACCT-3′,SORBS2-Rr:5′-TCCTATCACTAGAATAGCTG-3′;GAPDH-Fr:5′-CATGAGAAGTATGACAACAGCCT-3′,GAPDH-Rr:5′-AGTCCTTCCACGATACCAAAGT-3′.

### Tissue Microarrays (TMAs) and Immunohistochemistry (IHC)

Tissue microarrays (TMAs) were constructed with 231 pairs of primary ESCC tumor samples and matched normal esophageal epithelia as described previously [15], [16]. The ESCC specimens varied from pathological stage grade Ι to grade III from patients in the age group of 40–80 years. Standard streptavidin-biotin-peroxidase complex method was used for IHC staining. Briefly, TMA sections were deparaffinized, rehydrated and blocked by 10% normal goat serum at room temperature for 30 minutes. Sections were then incubated with anti-cortactin (CTTN) antibody (Abcam, Cambridge, UK) at a dilution of 1∶300 overnight at 4°C. After washed with TBS, the slides were then incubated with biotinylated goat anti-rabbit immunoglobulin at a concentration of 1∶100 for 30 min at 37°C. Three independent investigators semiquantitatively assessed CTTN positivity without prior knowledge of clinicopathologic data. Positive expression of CTTN in normal and malignant ESCC tissues was primarily a cytoplasm pattern. Because the intensity of CTTN staining within each tissue core was mostly homogeneous, the intensity of CTTN staining was semiquantitatively evaluated using the following criteria: strong positive (scored as 2+), dark brown staining in >50% of normal or malignant esophageal squamous cells completely obscuring cytoplasm; weak positive(1+), any lesser degree of brown staining appreciable in cell cytoplasm; absent (scored as 0), no appreciable staining in normal or malignant esophageal squamous cells. We classified strong positive expression as CTTN overexpression (+), weak positive and absent expression as CTTN no overexpression (–). Cases were accepted as being strongly positive only if the reviewers independently defined them as such.

### Statistical Analysis

Statistical analysis was done with the SPSS standard version 13.0 (SPSS Inc). The association between CTTN expression in tumor tissues and clinicopathologic characteristics such as age, gender, tumor cell differentiation, lymph nodes metastasis and pathological stage was analyzed using the chi-square test. Disease-specific survival (DSS) curves were calculated from the date of diagnosis to the date of death related to ESCC or the last date of follow-up. Survival curves were assessed by the Kaplan–Meier method and differences in survival times were compared by the log-rank test. Relative risks of cancer-related death associated with CTTN expression status and clinicopathologic variables including age, sex, tumor cell differentiation and lymph nodes metastasis were estimated by univariate analyses. Multivariate Cox analysis was performed on all variables that were detected to be significant on univariate level. Differences were considered significant when P value was less than 0.05.

## Results

### Identification of Differentially Expressed Genes between ESCC Tissues and Adjacent Normal Esophageal Epithelia

Gene expression profiles of pooled tumors (from 10 ESCCs) and their pooled normal counterparts were obtained by microarray analysis using Affymetrix human genome U133 plus 2.0 arrays covering 47,000 transcripts and variants. A total of 25,206 probe sets (transcripts) were present in ESCC tissues relative to their normal controls. We found that 1315 genes showed differentially expression levels. Among these genes, 549 up-regulated genes (41.7%, Table S1) and 766 down-regulated genes (58.3%, Table S2) were detected in tumor tissues compared with the expression profile of normal esophageal epithelia, using an arbitrary cutoff line of signal log ratio of ≥1.5 or ≤−1.5. The functional roles of 715 of these genes (340 up-regulated and 375 down-regulated) are known. The function of 302 differentially expressed genes (of 715, 42.24%) in tumor tissues were associated with cell metabolism (148 genes, Table S3), cell adhesion (90 genes, Table S4), and the immune response (64 genes, Table S5).

### Validation of Selected Genes using Semiquantitative PCR (RT-PCR)

**Figure 1:**
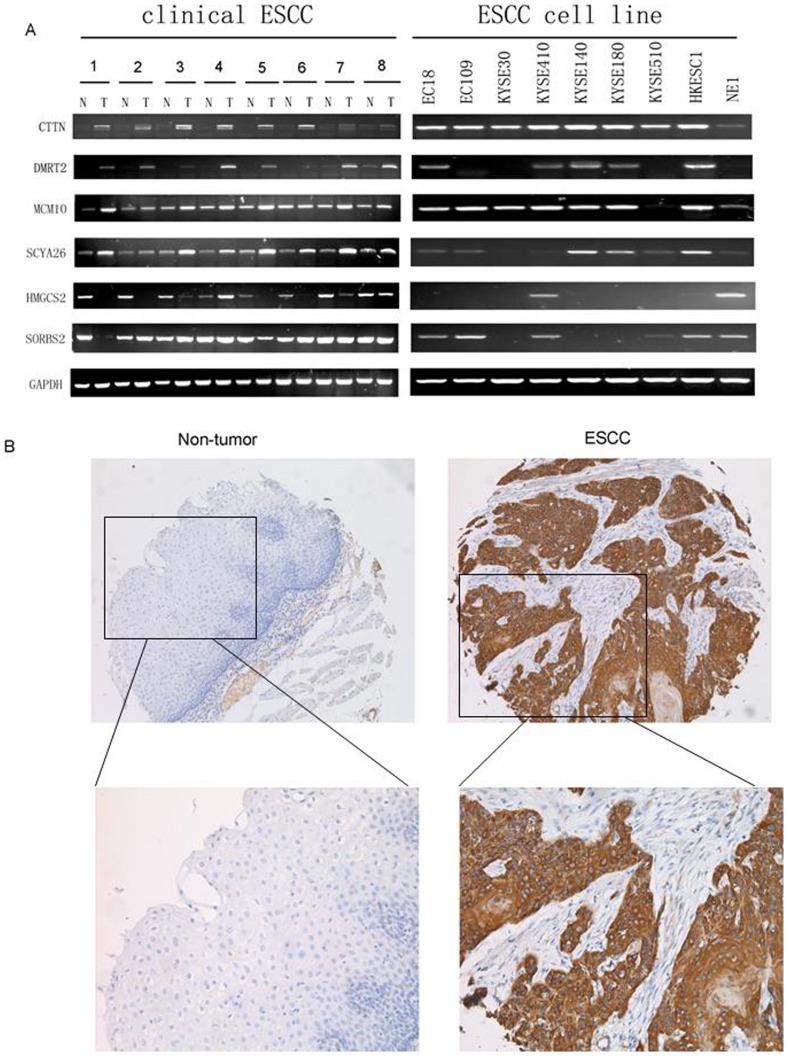
The differential expression of CTNN in ESCC. (A) Semiquantitative reverse transcription-PCR (RT-PCR) was applied to compare expression status of CTTN, DMRT2, MCM10, SCYA26, HMGCS2 and SORBS2 between 8 pairs of primary ESCC tumor samples and matched normal esophageal epithelia. GAPDH was used as an internal control. (B) Representative of CTNN expression in a pair of ESCC (right) and adjacent normal tissue (left) detected by immunostaining with anti-CTNN antibody (brown). The slide was counterstained with hematoxylin (Original magnification: upper ×100, bottom ×200).

To verify the microarray data, the expression levels of 6 randomly selected genes (4 up-regulated genes and 2 down-regulated genes) were examined by RT-PCR using the same RNA samples that were used for microarray analysis. The expression patterns of 6 randomly selected genes in tumor tissues detected by RT-PCR, except SORBS2, were fully consistent with those from the cDNA microarray results (Figure 1A).

### Evaluation of CTTN as a Prognostic Marker for ESCC

**Figure 2:**
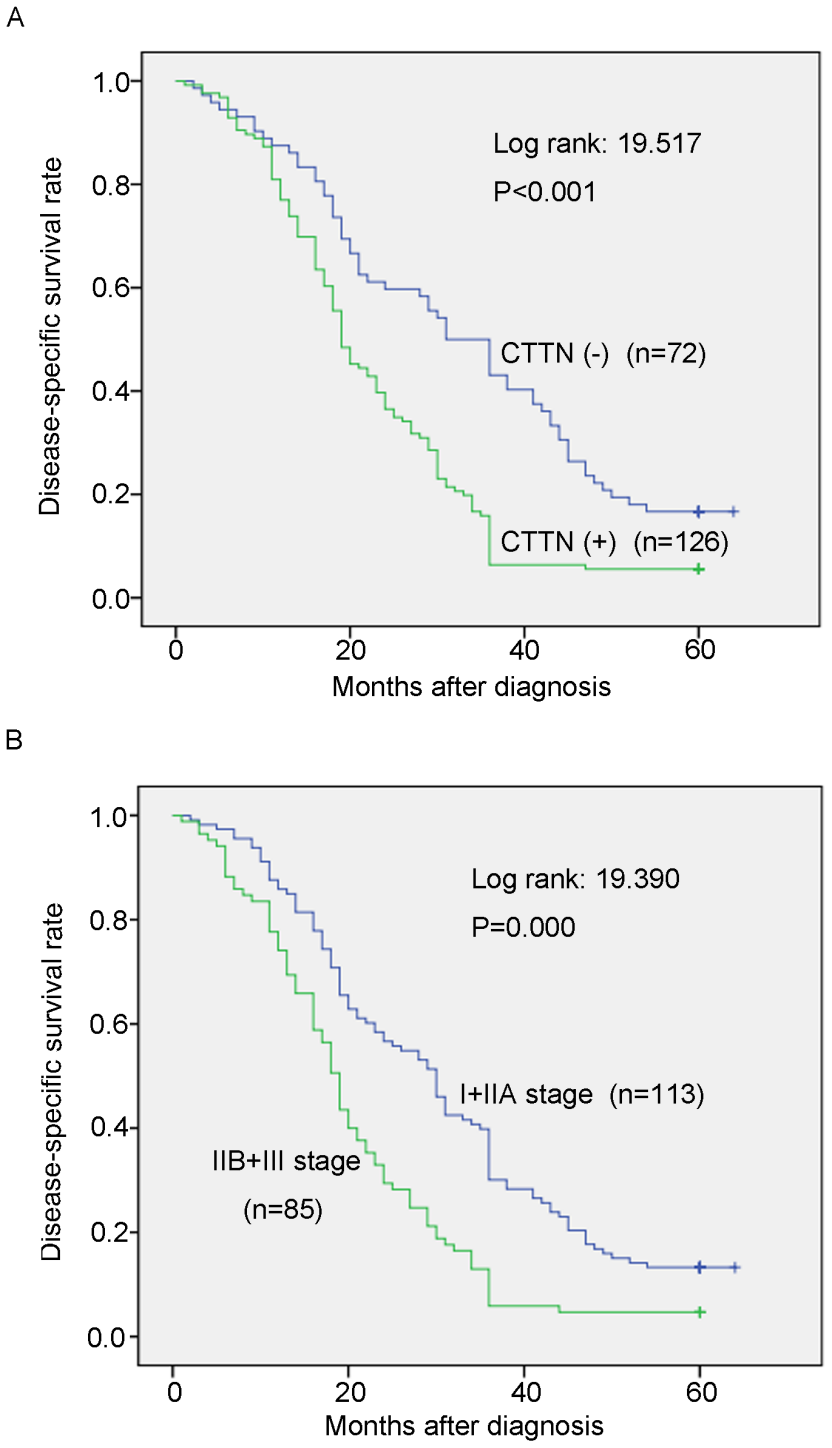
Kaplan-Meier plots for the Disease-specific survival rate of ESCC patients. (A) Kaplan-Meier plots for the Disease-specific survival (DSS) rate of ESCC patients with (n = 126, green line) or without (n = 72, blue line) CTNN overexpression. (B) Kaplan-Meier plots for the DSS rate of ESCC patients with pathologic stage I+IIA (n = 113, blue line) or IIB+III (n = 85, green line).

**Figure 3:**
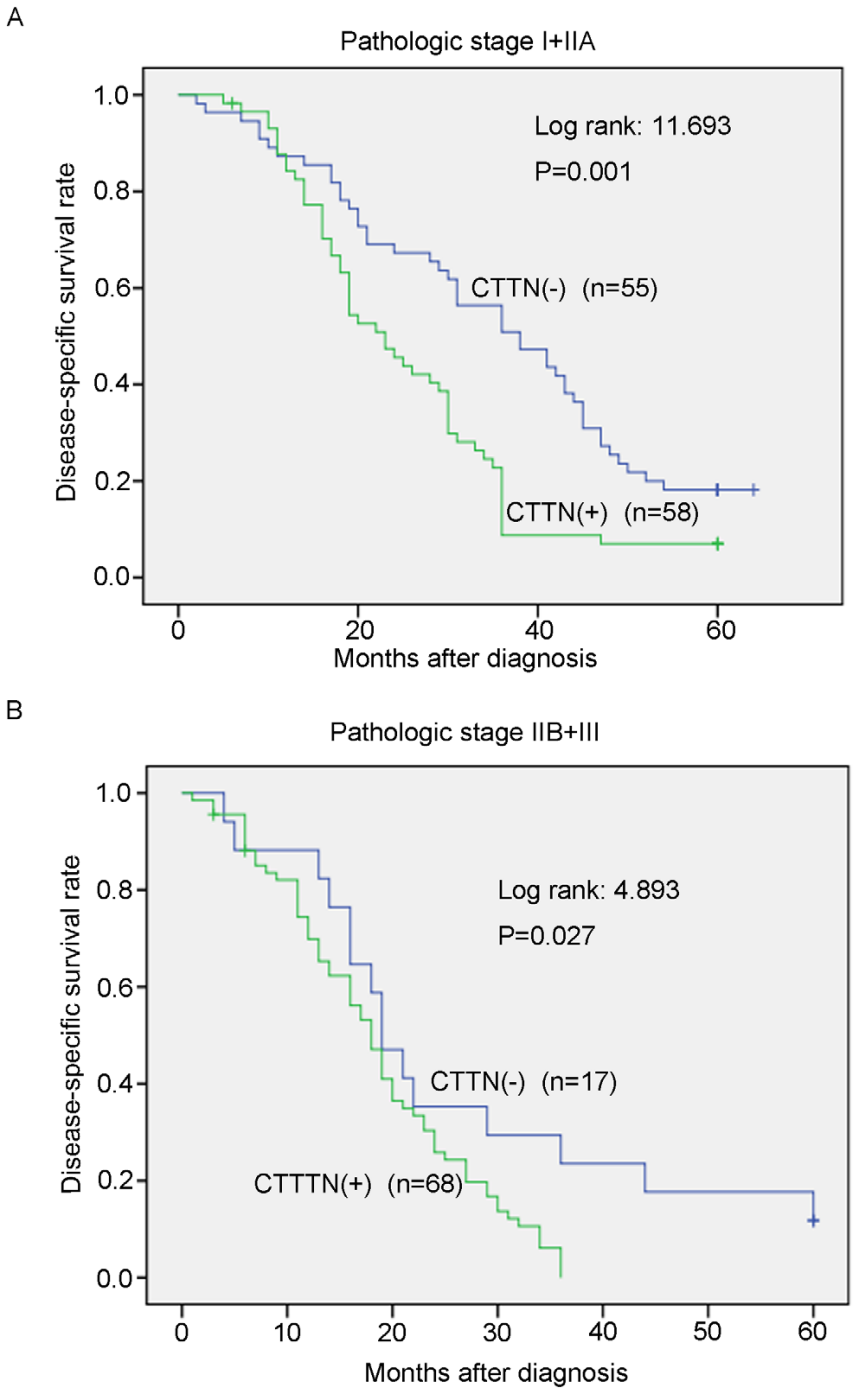
Kaplan-Meier plots for the DSS rate in ESCC patients subgrouped into pathologic stage I-IIA (A) and pathologic stage IIB-III (B) as differentiated by with (+) or without (−)-CTTN overexpression.

To evaluate the possibility of candidate overexpressed genes as biomarkers for ESCC, we carried out IHC staining with antibody for CTTN on tissue microarrays containing 231 pairs of ESCC tumor tissues and their adjacent normal esophageal epithelia. Informative IHC results were obtained from 198 ESCC cases. Non-informative samples included lost samples, inappropriately stained samples and samples with too few cells; such were not used as valid data. We observed the differential cytoplasm expression of CTTN between the primary ESCC tumors and their matched normal tissues (Figure 1B). The percentage of CTTN overexpression in informative ESCC tumor tissues was up to 63.6% (126/198), which was significantly higher than that in normal esophageal epithelia (26.8%, 53/198, *P*<0.001, Wilcoxon signed rank test, Figure 1B and Table 1). Of the 198 informative cases examined, the correlation between CTTN expression status and clinicopathologic features of ESCCs was detected. The results showed that CTTN overexpression was significantly associated with lymph node metastasis (P = 0.000) and pathological stage (P = 0.000). No significant difference was detected in age (P = 1.000), gender (P = 0.883) and tumor cell differentiation (P = 0.619, Table 2). Kaplan-Meier survival analysis showed that patients with CTTN (+) tumors (n = 126) have significantly worse survival rate than those with CTTN(–) tumors (n = 72) (P<0.001, log-rank: 19.517, Figure 2A). Meanwhile, Kaplan-Meier survival analysis also showed that patients with advanced pathologic stage (IIB+III; n = 85) have significantly worse survival rate than those with early pathologic stage (I+IIA; n = 113) (P = 0.000, log-rank: 19.390, Figure 2B). Furthermore, we examined the prognostic value of CTTN expression in ESCC patients with different pathologic stages. Patients with CTTN overexpression had significantly shorter disease-specific survival rate than those without CTTN overexpression in both I+IIA subgroup (n = 113, p = 0.001, Figure 3A) and IIB+III subgroup (n = 85, p = 0.027, Figure 3B), indicating that CTTN could be a valuable prognostic marker for ESCC. Furthermore, univariate analysis was used to evaluate associations between prognosis and several factors including age, sex, tumor cell differentiation, lymph nodes metastasis and CTTN status of ESCC tumors. The result showed that lymph nodes metastasis (P = 0.000), tumor cell differentiation (P = 0.001) and overexpression of CTTN in tumors (P = 0.000) were significant negative prognostic factors for ESCC patients (Table 3). Among those parameters, multivariate analyses using the Cox proportional hazard model revealed that overexpression of CTTN in ESCC tumors (P = 0.001), LN metastasis (P = 0.003) and tumor cell differentiation (P = 0.000) were independent prognostic factors for ESCC, respectively (Table 3). Collectively, our findings show that overexpression of CTTN in ESCC tumors independently predicts a poor prognosis for patients with ESCC.

**Table 1:** Expression of CTTN in ESCC and normal esophageal tissues.

Tissue	*n*	CTTN expressions			χ2	*P*
		Positive (*n*)	Negative (*n*)	%		
ESCC	198	126	72	63.6	27.164	<0.001*
Normal	198	53	145	26.8		

Note:*P<0.005.

**Table 2:** Association between CTTN over-expression and clinicopathologic characteristics of patients with ESCC (n = 198).

Clinicopathologic characteristics	N	CTTN expression no.(%)		χ2	*P* value
		Not overexpression	Over-expression		
Age(y)					
≤60	103	37(35.9)	66(64.1)	0.018	1.000
>60	95	35(36.8)	60(63.2)		
Gender					
Male	93	33(35.5)	60(64.5)	0.059	0.883
Female	105	39(37.1)	66(62.9)		
Tumor cell differentiation					
Well	27	9(33.3)	18(66.7)	0.959	0.619
Moderate	129	50(38.8)	79(61.2)		
Poor	42	13(31.0)	29(69.0)		
Lymph nodes metastasis					
N0	109	55(50.5)	54(49.5)	20.819	0.000*
N1	89	17(19.1)	72(80.9)		
Pathologic stage					
I+IIA	113	55(48.7)	58(51.3)	17.234	0.000*
IIB+III	85	17(20.0)	68(80.0)		

*P<0.05.

**Table 3:** Cox proportional hazard regression analyses for disease-specific survival.

Clinical features	*Univariate analysis*		*Multivariate analysis*	
	HR(95% CI)	P value	HR(95% CI)	P value
Age	1.211(0.924–1.587)	0.165		
Gender	1.135(0.864–1.490)	0.364		
LN metastasis	1.828(1.389–2.407)	**0.000** *	1.609(1.175–2.203)	**0.003** *
Differentiation	1.530(1.201–1.949)	**0.001** *	1.732(1.337–2.244)	**0.000** *
CTTN overregulation	1.987(1.443–2.738)	**0.000** *	1.820(1.296–2.555)	**0.001** *

CI = confidence interval; HR = hazard ration;

*Statistical significance(P<0.05) is shown in bold.

## Discussion

Esophageal squamous cell carcinoma (ESCC) is a highly aggressive cancer, being the fourth leading cause of death from cancer in China [17]. Due to the lack of reliable diagnostic techniques and specific symptoms for early stage detection, most patients have locally advanced of disseminated cancer at diagnosis when they swallow with difficulty or feel uncomfortable. Several tumor markers, such as carcinoembryonic antigen (CEA), squamous cell carcinoma antigen (SCC) and cytokeratin 19-fragment (CYFRA 21-1), are used in clinical diagnosis as well as in patient’s follow-up. EGFR is expressed in 33.3% of esophageal squamous cell carcinomas (ESCCs), Lapatinib can have a significant therapeutic effect against EGFR-expressing ESCC by inhibiting the growth of ESCC cells and augmenting Herceptin- and Cetuximab-mediated ADCC [18]. In fact, we haven’t discovered any useful tumor marker for the detection of ESCC at early stage and effective molecular-targeted therapies. A better understanding of molecular mechanism involved in occurrence and development of ESCC may lead to a more effective treatment for ESCC patients and find effective markers for the early detection and a better selection of adjuvant treatment modalities for appropriate ESCC patients. Determing differences in gene expression between ESCC tumor and normal tissues is essential to better understand this molecular mechanism.

Analysis of expression profiles by means of cDNA microarray is now widely used in various cancer cells [19]–[21]. A novel feature of the present study is that we used a microarray containing a very large number of probes to determine differential gene expression between ESCC tissues and matched normal esophageal epithelia by expression array. The result showed that 1315 genes were differentially expressed in ESCC. Among these genes, 549 were up-regulated and 766 were down-regulated. Of 715 differentially expressed genes with known function, 42.24% were associated with cell metabolism, cell adhesion and the immune response. In concordance with earlier reports, we identified genes, such as FASCIN (SNL), EGFR and ECRG4, were known to be differential expression in ESCC tissues and matched normal esophageal epithelia [18], [22]–[24]. In addition, we also discovered several ESCC-related genes which have not been previously reported. Some of differentially expressed genes may be useful as diagnostic markers, prognostic markers or novel therapeutic targets. In this study, we selected an up-regulated gene CTTN and examined its encoding protein (cortactin) expression status by tissue microarray analysis. Cortactin has been described as an actin-associated scaffolding protein, which binds and activates the actin related protein 2/3 complex, and has emerged as a central element connecting signaling pathways with cytoskeleton restructuring [25], [26]. Remodeling of the actin cytoskeleton has effects on cell migration, motility, and adhesion, as well as on tumor invasion and metastasis [27]. Overexpression of cortactin is associated with increased invasiveness of hepatocellular carcinoma cells [28].

Cortactin is overexpressed in many types of human cancers, including head and neck squamous carcinomas and colorectal, gastric, hepatocellular, breast and ovarian cancers [26], [29]. Previous reports showed that a significant correlation between CTTN overexpression and poor prognosis in osteosarcoma [30]. In this study, we evaluated the expression status of cortactin by using high-throughput TMA analysis. Our study has exhibited the potential value of CTTN in predicting patient survival in subgroups with early or advanced pathological stage, suggesting that overexpression of CTTN can be used as an independent factor for prognostic prediction of ESCC. However, more work need to be performed to further evaluate the CTTN gene for clinical applications to ESCC patients.

In summary, our cDNA microarray analysis detected differential gene expression profiles between ESCC tissues and normal esophageal epithelia and thereby provided valuable information for further study the molecular basis underlying the tumorigenesis of esophageal cancer. Better understanding the gene expression profiles of ESCC may lead to more effective management of ESCC by precise prognostic indicators and effective personalized therapy.

## Supporting Information

Figure S1 (TIF)

Table S1 **Up-regualted genes (≥1.5-fold) in ten cases of esophageal squamous cell carcinoma.**
(DOC)

Table S2 **Down-regulated genes (≥1.5-fold) in ten cases of esophageal squamous cell carcinoma.**
(DOC)

Table S3 **List of 148 genes associated with cell metabolism.**
(DOC)

Table S4 **List of 90 genes associated with cell adhesion.**
(DOC)

Table S5 **List of 64 genes associated with immune response.**
(DOC)
