# Supplementary figures and images for: Genome-Wide Gene Expression Profile Analyses Identify CTTN as a Potential Prognostic Marker in Esophageal Cancer
Source: PLoS One. 2014 Feb 14;9(2):e88918. doi: 10.1371/journal.pone.0088918 (PMC3925182; doi:10.1371/journal.pone.0088918)

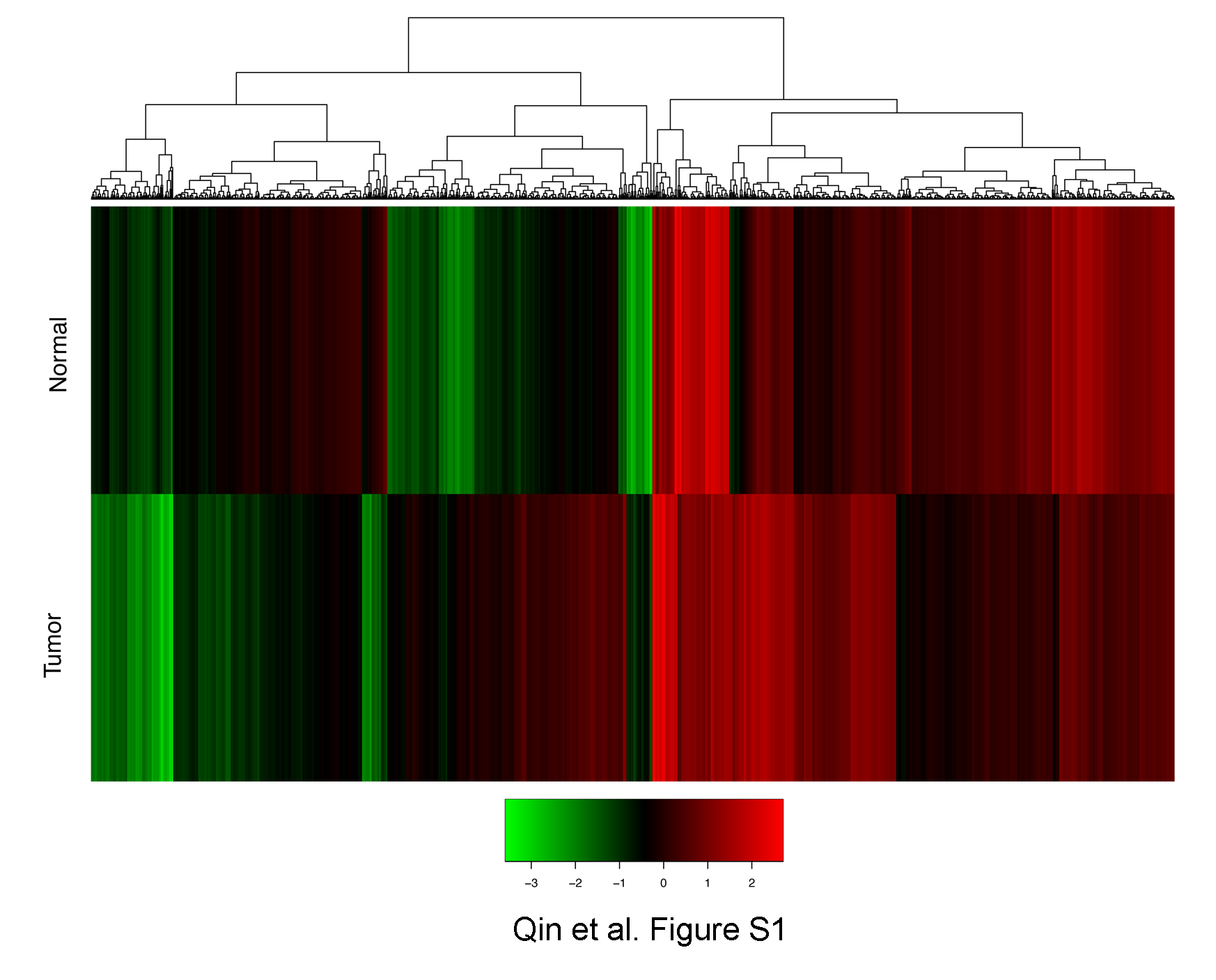

Supplement: Figure S1 — (TIF) [file pone.0088918.s001.tif]
