# Supplementary material for: Genome-Wide Gene Expression Profile Analyses Identify CTTN as a Potential Prognostic Marker in Esophageal Cancer
Source: PLoS One. 2014 Feb 14;9(2):e88918. doi: 10.1371/journal.pone.0088918 (PMC3925182; doi:10.1371/journal.pone.0088918)
Supplement: Table S1 — Up-regualted genes (≥1.5-fold) in ten cases of esophageal squamous cell carcinoma. (DOC) [file pone.0088918.s002.doc]

Table S1. Up-regualted genes (≥1.5-fold) in ten cases of esophageal squamous cell carcinoma

| **Gene symbol** | **Gene name** | **Probe set ID** | **Fold-**  **change** | **GenBank no.** |
| --- | --- | --- | --- | --- |
| GAGE7B | G antigen 7B | 206640_x_at | 7.4 | NM_001477.1 |
| GAGE2 | G antigen 2 | 207739_s_at | 7.1 | NM_001472.1 |
| GAGE4 | G antigen 4 | 207086_x_at | 6.8 | NM_001474.1 |
| COL10A1 | collagen, type X, alpha 1(Schmid metaphyseal chondrodysplasia) | 217428_s_at | 6.3 | X98568 |
| MMP13 | matrix metalloproteinase 13 (collagenase 3) | 205959_at | 6.2 | NM_002427.2 |
| IBSP | Integrin-binding sialoprotein (bone sialoprotein, bone sialoprotein II) | 236028_at | 6.1 | BE466675 |
| GAGE3 | G antigen 3 | 207663_x_at | 6 | NM_001473.1 |
| COL11A1 | collagen, type XI, alpha 1 | 37892_at | 6 | J04177 |
| CALB1 | calbindin 1, (28kD) | 205626_s_at | 5.9 | NM_004929.2 |
| MMP10 | matrix metalloproteinase 10 (stromelysin 2) | 205680_at | 5.3 | NM_002425.1 |
| ITGB6 | integrin, beta 6 | 208084_at | 5.3 | NM_000888.3 |
| LGR5 | leucine-rich repeat-containing G protein-coupled receptor 5 | 213880_at | 5.3 | AL524520 |
| DSPG3 | dermatan sulphate proteoglycan 3 | 206439_at | 5.2 | NM_004950.2 |
| KRTHB1 | keratin, hair, basic, 1 | 213711_at | 5.2 | NM_002281.1 |
| PNLIP | pancreatic lipase | 205912_at | 5.1 | NM_000936.1 |
| POLE4 | Polymerase (DNA-directed), epsilon 4 (p12 subunit) | 1565329_at | 5.1 | AY034104.1 |
| COL11A1 | collagen, type XI, alpha 1 | 204320_at | 5 | NM_001854.1 |
| LIPF | lipase, gastric | 206334_at | 5 | NM_004190.1 |
| MYL2 | myosin, light chain 2, regulatory, cardiac, slow | 209742_s_at | 5 | AF020768.1 |
| TNNC1 | troponin C type 1 (slow) | 209904_at | 5 | AF020769.1 |
| PGC | progastricsin (pepsinogen C) | 1555236_a_at | 5 | BC042578.1 |
| MMP3 | matrix metalloproteinase 3 (stromelysin 1, progelatinase) | 205828_at | 4.9 | NM_002422.2 |
| DIAPH3 | diaphanous homolog 3 (Drosophila) | 232596_at | 4.9 | AL354829 |
| GAGE6 | G antigen 6 | 208155_x_at | 4.8 | NM_001476.1 |
| DHRS2 | dehydrogenase/reductase (SDR family) member 2 | 214079_at | 4.8 | AK000345.1 |
| AKAP3 | A kinase (PRKA) anchor protein 3 | 207344_at | 4.7 | NM_006422.1 |
| HMGIC | high-mobility group (nonhistone chromosomal) protein isoform I-C | 208025_s_at | 4.7 | NM_003483.2 |
| DMRT1 | doublesex and mab-3 related transcription factor 1 | 220493_at | 4.7 | NM_021951.1 |
| GRP | gastrin-releasing peptide | 206326_at | 4.6 | NM_002091.1 |
| MMP8 | matrix metalloproteinase 8 (neutrophil collagenase) | 207329_at | 4.6 | NM_002424.1 |
| EPHB2 | protein-tyrosine kinase EPHB2v | 209589_s_at | 4.6 | AF025304.1 |
| MMP1 | matrix metalloproteinase 1 (interstitial collagenase) | 204475_at | 4.5 | NM_002421.2 |
| PGC | progastricsin (pepsinogen C) | 205261_at | 4.5 | NM_002630.1 |
| CSRP3 | cysteine and glycine-rich protein 3 (cardiac LIM protein) | 205553_s_at | 4.5 | NM_003476.1 |
| LHX2 | LIM homeobox protein 2 | 206140_at | 4.5 | NM_004789.1 |
| INHBA | inhibin, beta A (activin A, activin AB alpha polypeptide) | 210511_s_at | 4.5 | M13436.1 |
| PLAC1 | placenta-specific 1 | 219702_at | 4.5 | NM_021796.1 |
| CDSN | corneodesmosin | 206193_s_at | 4.4 | NM_001264.1 |
| CST1 | cystatin SN | 206224_at | 4.4 | NM_001898.1 |
| GAGE7 | G antigen 7 | 208235_x_at | 4.4 | NM_021123.1 |
| EGFR | epidermal growth factor receptor precursor | 211607_x_at | 4.3 | U48722.1 |
| DLX2 | distal-less homeo box 2 | 207147_at | 4.2 | NM_004405.2 |
| CXCL5 | chemokine (C-X-C motif) ligand 5 | 214974_x_at | 4.2 | AK026546.1 |
| CAPZA1 | capping protein (actin filament) muscle Z-line, alpha 1 | 217392_at | 4.2 | AL024493 |
| CTRB1 | chymotrypsinogen B1 | 205971_s_at | 4.1 | NM_001906.1 |
| SLC21A8 | solute carrier family 21 (organic anion transporter), member 8 | 206354_at | 4.1 | NM_019844.1 |
| HOXD13 | homeo box D13 | 207397_s_at | 4.1 | NM_000523.1 |
| PGA3 PGA4 PGA5 | pepsinogen 5, group I (pepsinogen A) pepsinogen 3, group I (pepsinogen A) pepsinogen 4, group I (pepsinogen A) | 213265_at | 4.1 | AI570199 |
| FGF19 | fibroblast growth factor 19 | 223761_at | 4.1 | AF110400.1 |
| MMP11 | matrix metallopeptidase 11 (stromelysin 3) | 203876_s_at | 4 | AI761713 |
| PAP | pancreatitis-associated protein | 205815_at | 4 | NM_002580.1 |
| WT1 | Wilms tumor 1 | 206067_s_at | 4 | NM_024426.1 |
| MYL1 | Myosin light chain | 209888_s_at | 4 | M_20643.1 |
| CHRNA9 | cholinergic receptor, nicotinic, alpha polypeptide 9 | 221107_at | 4 | NM_017581.1 |
| IGSF9 | immunoglobulin superfamily, member 9 | 229276_at | 4 | AB037776 |
| GPR39 | G protein-coupled receptor 39 | 212909s_at | 3.9 | AL567376 |
| SFRP4 | secreted frizzled-related protein 4 | 204051_s_at | 3.8 | AW089415 |
| SCYB5 | Small inducible cytokine subfamily B, number 5 | 215101_s_at | 3.8 | BG166705 |
| BCL7A | B-cell CLL/lymphoma 7A | 203796_s_at | 3.7 | AI950380 |
| MMP11 | matrix metalloproteinase 11 (stromelysin 3) | 203878_s_at | 3.7 | NM_005940.2 |
| STG | STG protein | 221100_at | 3.7 | NM_014070.1 |
| SOST | SOST | 223869_at | 3.7 | AF331844.1 |
| ZNF7 | zinc finger protein 7 | 1559980_at | 3.7 | AI862153 |
| CCNYL2 | cyclin Y-like 2 | 1569878_at | 3.7 | BC039000.1 |
| CCA1 | cyclin A1 | 205899_at | 3.6 | NM_003914.1 |
| PAEP | progestagen-associated endometrial protein (placental protein 14, pregnancy-associated endometrial alpha-2-globulin, alpha uterine protein) | 206859_s_at | 3.6 | NM_002571.1 |
| LHX1 | LIM homeobox protein 1 | 206230_at | 3.5 | NM_005568.1 |
| ADAM12 | a disintegrin and metalloproteinase domain 12 (meltrin alpha) | 202952_s_at | 3.4 | NM_003474.2 |
| SPINK1 | serine protease inhibitor, Kazal type 1 | 206239_s_at | 3.4 | NM_003122.1 |
| COL1A1 | collagen, type I, alpha 1 | 202310_s_at | 3.3 | K01228.1 |
| CSF2 | colony stimulating factor 2 (granulocyte-macrophage) | 210229_s_at | 3.3 | M11734.1 |
| WISP1 | WNT1 inducible signaling pathway protein 1 | 211312_s_at | 3.3 | AB034725.1 |
| SFRP4 | secreted frizzled-related protein 4 | 204052_s_at | 3.2 | NM_003014.2 |
| IL11 | interleukin 11 | 206924_at | 3.2 | NM_000641.1 |
| HUMCYT2A | cytokeratin 2 | 207878_at | 3.2 | NM_015848.1 |
| PTHLH | parathyroid hormone-like hormone | 211756_at | 3.2 | BC005961.1 |
| MFAP2 | microfibrillar-associated protein 2 | 203417_at | 3.1 | NM_017459.1 |
| CA9 | carbonic anhydrase IX | 205199_at | 3.1 | NM_001216.1 |
| SCYB5 | small inducible cytokine subfamily B (Cys-X-Cys), member 5 (epithelial-derived neutrophil-activating peptide 78) | 207852_at | 3.1 | NM_002994.1 |
| LIBC | lost in inflammatory breast cancer tumor suppressor protein | 210861_s_at | 3.1 | AF143679.1 |
| POP3 | popeye protein 3 | 219926_at | 3.1 | NM_022361.1 |
| LAMC2 | laminin, gamma 2 (nicein (100kD), kalinin (105kD), BM600 (100kD), Herlitz junctional epidermolysis bullosa)) | 202267_at | 3 | NM_005562.1 |
| LOXL2 | lysyl oxidase-like 2 | 202997_s_at | 3 | BE251211 |
| NRG1 | neuregulin 1 | 206237_s_at | 3 | NM_013957.1 |
| PTHLH | parathyroid hormone-like hormone | 206300_s_at | 3 | NM_002820.1 |
| SPP1 | secreted phosphoprotein 1 (osteopontin, bone sialoprotein I, early T-lymphocyte activation 1) | 209875_s_at | 3 | M83248.1 |
| PTPRR | protein tyrosine phosphatase, receptor type, R | 210675_s_at | 3 | U77917.1 |
| TREX2 | clone T2P4 3-5 exonuclease TREX2 | 211788_s_at | 3 | AF319573.1 |
| HOXC13 | homeo box C13 | 219832_s_at | 3 | NM_017410.1 |
| GDF15 | Growth differentiation factor 15 | 229868_s_at | 3 | AA129612 |
| POSTN | periostin, osteoblast specific factor | 1555778_a_at | 3 | AY140646.1 |
| LAMA3 | laminin, alpha 3 (nicein (150kD), kalinin (165kD), BM600 (150kD), epilegrin) | 203726_s_at | 2.9 | NM_000227.1 |
| ACTA1 | actin, alpha 1, skeletal muscle | 203872_at | 2.9 | NM_001100.2 |
| CYP26A1 | cytochrome P450, subfamily XXVIA, polypeptide 1 | 206424_at | 2.9 | NM_000783.1 |
| HEP27 | short-chain alcohol dehydrogenase family member | 206463_s_at | 2.9 | NM_005794.1 |
| LGR7 | leucine-rich repeat-containing G protein-coupled receptor 7 | 1552715_a_at | 2.9 | NM_021634.1 |
| MME | membrane metallo-endopeptidase | 203434_s_at | 2.8 | AI433463 |
| GAD1 | glutamate decarboxylase 1 (brain, 67kD) | 206670_s_at | 2.8 | NM_013445.1 |
| MAGEA1 | melanoma antigen, family A, 1 (directs expression of antigen MZ2-E) | 207325_x_at | 2.8 | NM_004988.1 |
| IL8 | interleukin 8 C-terminal variant | 211506_s_at | 2.8 | AF043337.1 |
| KIAA1622 | KIAA1622 protein | 220673_s_at | 2.8 | NM_020958.1 |
| FGFBP2 | fibroblast growth factor binding protein 2 | 223836_at | 2.8 | AB021123.1 |
| HTATIP2 | HIV-1 Tat interactive protein 2, 30kDa | 229102_at | 2.8 | BG153401 |
| FREM2 | FRAS1 related extracellular matrix protein 2 | 230964_at | 2.8 | N66307 |
| LOC118430 | small breast epithelial mucin | 1553602_at | 2.8 | NM_058173.1 |
| WNT2 | wingless-type MMTV integration site family member 2 | 205648_at | 2.7 | NM_003391.1 |
| GABRR1 | gamma-aminobutyric acid (GABA) receptor, rho 1 | 206525_at | 2.7 | NM_002042.1 |
| ST16 | suppression of tumorigenicity 16 (melanoma differentiation) | 206569_at | 2.7 | NM_006850.1 |
| PLAU | plasminogen activator, urokinase | 211668_s_at | 2.7 | K03226.1 |
| HOXC10 | homeo box C10 | 218959_at | 2.7 | NM_017409.1 |
| COL5A1 | collagen, type V, alpha 1 | 203325_s_at | 2.6 | AI130969 |
| CCNF | cyclin F | 204826_at | 2.6 | NM_001761.1 |
| ISG15 | interferon-stimulated protein, 15 kDa | 205483_s_at | 2.6 | NM_005101.1 |
| COL5A3 | collagen, type V, alpha 3 | 218975_at | 2.6 | NM_015719.1 |
| MMP12 | matrix metalloproteinase 12 (macrophage elastase) | 204580_at | 2.5 | NM_002426.1 |
| SERPINH1 | serine (or cysteine) proteinase inhibitor, clade H (heat shock protein 47), member 1 | 207714_s_at | 2.5 | NM_004353.1 |
| TREM2 | triggering receptor expressed on myeloid cells 2 | 219725_at | 2.5 | NM_018965.1 |
| COL5A2 | collagen, type V, alpha 2 | 221730_at | 2.5 | NM_000393.1 |
| GPR39 | G protein-coupled receptor 39 | 229105_at | 2.5 | AV717094 |
| FBN2 | fibrillin 2 (congenital contractural arachnodactyly) | 203184_at | 2.4 | NM_001999.2 |
| MMP9 | matrix metalloproteinase 9 (gelatinase B, 92kD gelatinase, 92kD type IV collagenase) | 203936_s_at | 2.4 | NM_004994.1 |
| WISP1 | WNT1 inducible signaling pathway protein 1 | 206796_at | 2.4 | NM_003882.1 |
| OSF-2p1 | osf-2 mRNA for osteoblast specific factor 2 | 210809_s_at | 2.4 | D13665.1 |
| CTAG1A CTAG1B | cancer/testis antigen 1B cancer/testis antigen 1A | 211674_x_at | 2.4 | AF038567.1 |
| HOXD11 | homeo box D11 | 214604_at | 2.4 | NM_021192.1 |
| MAGEA6 | melanoma antigen family A, 6 | 214612_x_at | 2.4 | U10691 |
| LOC51086 | putative protein-tyrosine kinase | 220415_at | 2.4 | NM_015978.1 |
| GCAP | guanylate cyclase activating protein | 1255_g_at | 2.3 | L36861 |
| G1P3 | interferon, alpha-inducible protein (clone IFI-6-16) | 204415_at | 2.3 | NM_022873.1 |
| T1A2 | lung type-I cell membrane-associated glycoprotein | 204879_at | 2.3 | NM_006474.1 |
| PLAU | plasminogen activator, urokinase | 205479_s_at | 2.3 | NM_002658.1 |
| CCND2 | cyclin D2 | 200951_s_at | 2.2 | AW026491 |
| UCHL1 | ubiquitin carboxyl-terminal esterase L1 (ubiquitin thiolesterase) | 201387_s_at | 2.2 | NM_004181.1 |
| MSX2 | msh homeobox 2 | 205555_s_at | 2.2 | D31771.1 |
| NRG1 | neuregulin 1 | 206343_s_at | 2.2 | NM_013959.1 |
| EPHA7 | EphA7 | 206852_at | 2.2 | NM_004440.1 |
| DSC1 | desmocollin 1 | 207324_s_at | 2.2 | NM_004948.2 |
| ESM1 | endothelial cell-specific molecule 1 | 208394_x_at | 2.2 | NM_007036.2 |
| FAP | fibroblast activation protein, alpha | 209955_s_at | 2.2 | U76833.1 |
| CXCL11 | chemokine (C-X-C motif) ligand 11 | 210163_at | 2.2 | AF030514.1 |
| DLX5 | distal-less homeo box 5 | 213707_s_at | 2.2 | NM_005221.3 |
| FN1 | fibronectin 1 | 214702_at | 2.2 | AJ276395.1 |
| HOXB7 | homeobox B7 | 216973_s_at | 2.2 | S49765.1 |
| HT013 | uncharacterized hypothalamus protein HT013 | 219961_s_at | 2.2 | NM_018474.1 |
| CPX1 | metallocarboxypeptidase CPX-1 | 227860_at | 2.2 | NM_019609.1 |
| GPR126 | G protein-coupled receptor 126 | 233887_at | 2.2 | AL033377 |
| ENAH | enabled homolog (Drosophila) | 1553672_at | 2.2 | M_145240.1 |
| CYP27C1 | cytochrome P450, family 27, subfamily C, polypeptide 1 | 1568868_at | 2.2 | BC039307.1 |
| SLC2A1 | solute carrier family 2 (facilitated glucose transporter), member 1 | 201250_s_at | 2.1 | NM_006516.1 |
| EGFR | epidermal growth factor receptor (avian erythroblastic leukemia viral (v-erb-b) oncogene homolog) | 201984_s_at | 2.1 | NM_005228.1 |
| MMP14 | matrix metallopeptidase 14 (membrane-inserted) | 202827_s_at | 2.1 | AU149305 |
| LOXL2 | lysyl oxidase-like 2 | 202998_s_at | 2.1 | NM_002318.1 |
| RBP1 | retinol-binding protein 1, cellular | 203423_at | 2.1 | NM_002899.2 |
| MATN3 | matrilin 3 | 206091_at | 2.1 | NM_002381.2 |
| ITGB6 | integrin, beta 6 | 208083_s_at | 2.1 | NM_000888.3 |
| TRIO | triple functional domain (PTPRF interacting) | 208178_x_at | 2.1 | NM_007118.1 |
| CDKN2A | cyclin-dependent kinase inhibitor 2A (melanoma, p16, inhibits CDK4) | 209644_x_at | 2.1 | U38945.1 |
| IGFBP3 | insulin-like growth factor binding protein 3 | 212143_s_at | 2.1 | BF340228 |
| DIRAS3 | DIRAS family, GTP-binding RAS-like 3 | 215506_s_at | 2.1 | AK021882.1 |
| PLEK2 | pleckstrin 2 (mouse) homolog | 218644_at | 2.1 | NM_016445.1 |
| NOX4 | NADPH oxidase 4 | 219773_at | 2.1 | NM_016931.1 |
| PRSS21 | protease, serine, 21 (testisin) | 220051_at | 2.1 | NM_006799.1 |
| TRAG3 | taxol resistance associated gene 3 | 220445_s_at | 2.1 | NM_004909.1 |
| SESN3 | sestrin 3 | 235683_at | 2.1 | BF685808 |
| TOB3 | AAA-ATPase TOB3 | 1552641_s_at | 2.1 | NM_031921.1 |
| ZMYND11 | zinc finger, MYND domain containing 11 | 1554158_at | 2.1 | BC034784.1 |
| SERPINE1 | serpin peptidase inhibitor, clade E (nexin, plasminogen activator inhibitor type 1), member 1 | 202627_s_at | 2 | AL574210 |
| SCYA3 | small inducible cytokine A3 (homologous to mouse Mip-1a) | 205114_s_at | 2 | NM_002983.1 |
| BMP1 | bone morphogenetic protein 1 | 205574_x_at | 2 | NM_001199.1 |
| CDKN2A | cyclin-dependent kinase inhibitor 2A (melanoma, p16, inhibits CDK4) | 207039_at | 2 | NM_000077.1 |
| K6HF | cytokeratin type II | 207065_at | 2 | NM_004693.1 |
| CDH11 | cadherin 11, type 2, OB-cadherin (osteoblast) | 207172_s_at | 2 | NM_001797.1 |
| CST2 | cystatin SA | 208555_x_at | 2 | NM_001322.1 |
| OLR1 | oxidized low density lipoprotein (lectin-like) receptor 1 | 210004_at | 2 | AF035776.1 |
| OASL | 2-5oligoadenylate synthetase-related protein p30 | 210797_s_at | 2 | AF063612.1 |
| H174 | putative alpha chemokine | 211122_s_at | 2 | AF002985.1 |
| VCAN | versican | 211571_s_at | 2 | D32039.1 |
| HOXA10 | homeobox A10 | 213150_at | 2 | BF792917 |
| IMP-2 | IGF-II mRNA-binding protein 2 | 218847_at | 2 | NM_006548.1 |
| DKK2 | dickkopf (Xenopus laevis) homolog 2 | 219908_at | 2 | NM_014421.1 |
| PYY2 | peptide YY, 2 (seminalplasmin) | 220209_at | 2 | NM_021093.1 |
| ZCYTO10 | four alpha helix cytokine | 224071_at | 2 | AF224266.1 |
| GDA | guanine deaminase | 224209_s_at | 2 | AF019638.1 |
| MRGX3 | G protein-coupled receptor MRGX3 | 1553293_at | 2 | NM_054031.1 |
| SNL | singed (Drosophila)-like (sea urchin fascin homolog like) | 201564_s_at | 1.9 | NM_003088.1 |
| COL1A2 | collagen, type I, alpha 2 | 202404_s_at | 1.9 | NM_000089.1 |
| FSTL3 | follistatin-like 3 glycoprotein | 203592_s_at | 1.9 | NM_005860.1 |
| SGNE1 | secretory granule, neuroendocrine p rotein 1 (7B2protein) | 203889_at | 1.9 | NM_003020.1 |
| COL17A1 | collagen, type XVII, alpha 1 | 204636_at | 1.9 | NM_000494.1 |
| IFIT4 | interferon-induced protein with tetratricopeptide repeats 4 | 204747_at | 1.9 | NM_001549.1 |
| CHEK1 | CHK1 (checkpoint, S.pombe) homolog | 205394_at | 1.9 | NM_001274.1 |
| CYP27B1 | cytochrome P450, subfamily XXVIIB | 205676_at | 1.9 | NM_000785.1 |
| SERPINB7 | serine (or cysteine) proteinase inhibitor, cladeB (ovalbumin), member 7 | 206421_s_at | 1.9 | NM_003784.1 |
| FLJ20132 BEST2 修改 | hypothetical protein FLJ20132 bestrophin 2 | 207432_at | 1.9 | NM_017682.1 |
| KIAA0230 PXDN | Melanoma associated gene peroxidasin homolog | 212013_at | 1.9 | D86983.1 |
| TNNT1 | troponin T1, skeletal, slow | 213201_s_at | 1.9 | AJ011712 |
| BGN | biglycan | 213905_x_at | 1.9 | AA845258 |
| GAL | galanin | 214240_at | 1.9 | AL556409 |
| MAGEA2 | melanoma antigen, family A, 2 | 214603_at | 1.9 | U82671 |
| LOX7 | lysyl oxidase (LOX) gene, exon 7 | 215446_s_at | 1.9 | L16895 |
| TONDU | vestigial like 1 | 215729_s_at | 1.9 | BE542323 |
| TMEPAI | transmembrane, prostate androgen induced RNA | 217875_s_at | 1.9 | NM_020182.1 |
| DLL3 | delta-like 3 protein precursor | 219537_x_at | 1.9 | NM_016941.1 |
| CLSP | calmodulin-like skin protein | 220414_at | 1.9 | NM_017422.2 |
| PRO1912 | exocyst complex component 5 | 224253_at | 1.9 | AF118083.1 |
| DCBLD1 | discoidin, CUB and LCCL domain containing 1 | 1553768_a_at | 1.9 | NM_173674.1 |
| TGFBI | transforming growth factor, beta-induced, 68kD | 201506_at | 1.8 | NM_000358.1 |
| LY6E | lymphocyte antigen 6 complex, locus E | 202145_at | 1.8 | NM_002346.1 |
| CDC20 | cell division cycle 20 | 202870_s_at | 1.8 | NM_001255.1 |
| IFIT1 | interferon-induced protein with tetratricopeptide repeats 1 | 203153_at | 1.8 | NM_001548.1 |
| STC2 | stanniocalcin 2 | 203438_at | 1.8 | AI435828 |
| HMG4 | high-mobility group (nonhistone chromosomal)protein 4 | 203744_at | 1.8 | NM_005342.1 |
| SCYB10 | interferon gamma-induced precursor | 204533_at | 1.8 | NM_001565.1 |
| FST | follistatin isoform FST344 precursor | 204948_s_at | 1.8 | NM_013409.1 |
| KRT17 | keratin 17 | 205157_s_at | 1.8 | NM_000422.1 |
| IL1RL1 | interleukin 1 receptor-like 1 | 207526_s_at | 1.8 | NM_003856.1 |
| LAMB3 | laminin S B3 chain | 209270_at | 1.8 | L25541.1 |
| CDT1 | DNA replication factor | 209832_s_at | 1.8 | AF321125.1 |
| C20ORF1 | restricted expressed proliferation associated protein 100 | 210052_s_at | 1.8 | AF098158.1 |
| UPK1B | human uroplakin Ib | 210065_s_at | 1.8 | AB002155.1 |
| IGFBP1 | insulin-like growth factor binding protein 3 | 210095_s_at | 1.8 | M31159.1 |
| OG12 | homeodomain protein | 210135_s_at | 1.8 | AF022654.1 |
| PPFIA1 | LAR-interacting protein 1a | 210236_at | 1.8 | U22815.1 |
| PPBP | pro-platelet basic protein (includes platelet basic protein, beta-thromboglobulin | 214146_s_at | 1.8 | R64130 |
| SLC7A8 | solute carrier family 7 (cationic amino acid transporter, y+ system), member 8 | 216092_s_at | 1.8 | AL365347.1 |
| ZNF639 | zinc finger protein 639 | 218413_s_at | 1.8 | NM_016331.1 |
| LOC57333 | reticulocalbin 3, EF-hand calcium binding domain | 219102_at | 1.8 | NM_020650.1 |
| PRV1 | polycythemia rubra vera 1， cell surface receptor | 219669_at | 1.8 | NM_020406.1 |
| EN1 | engrailed homolog 1 | 220559_at | 1.8 | NM_001426.2 |
| NEFL | neurofilament, light polypeptide (68kD) | 221805_at | 1.8 | AL537457 |
| ZIC2 | zinc finger protein of cerebellum ZIC2 | 223642_at | 1.8 | AF193855.1 |
| SCYA26 | thymic stroma chemokine-1 precursor | 223710_at | 1.8 | AF096296.1 |
| FSP-2 | testis-specific calcium-binding proteinCBP86-IV | 224279_s_at | 1.8 | AF295039.1 |
| MTBP | MDM2 (mouse double minute 2)-binding protein, 104kD | 233211_at | 1.8 | AK022122.1 |
| ZNF114 | zinc finger protein 114 | 1552946_at | 1.8 | NM_153608.1 |
| DSG2 | desmoglein 2 preproprotein | 1553105_s_at | 1.8 | NM_001943.1 |
| FLJ33951 | ATP-binding cassette, sub-family A (ABC1), member 13 | 1553604_at | 1.8 | NM_152701.1 |
| ACP1 | acid phosphatase 1, soluble | 201629_s_at | 1.7 | BE872974 |
| FOXM1 | forkhead box M1 | 202580_x_at | 1.7 | NM_021953.1 |
| CDC45L | CDC45 (cell division cycle 45, S.cerevisiae,homolog)-like | 204126_s_at | 1.7 | NM_003504.1 |
| APOC1 | apolipoprotein C-I precursor | 204416_x_at | 1.7 | NM_001645.2 |
| PDGFRL | platelet-derived growth factor receptor-like | 205226_at | 1.7 | NM_006207.1 |
| DUSP9 | dual specificity phosphatase | 205777_at | 1.7 | NM_001395.1 |
| TDO2 | tryptophan 2,3-dioxygenase | 205943_at | 1.7 | NM_005651.1 |
| ZIC1 | Zic family member 1 (odd-paired Drosophilahomolog) | 206373_at | 1.7 | NM_003412.1 |
| SYCP2 | synaptonemal complex protein 2 | 206546_at | 1.7 | NM_014258.1 |
| VIP | vasoactive intestinal peptide | 206577_at | 1.7 | NM_003381.1 |
| CDK6 | cyclin-dependent kinase 6 | 207143_at | 1.7 | NM_001259.1 |
| PF4V1 | platelet factor 4 variant 1 | 207815_at | 1.7 | NM_002620.1 |
| STK6 | serinethreonine kinase 6 | 208080_at | 1.7 | NM_003158.1 |
| UGT8 | UDP glycosyltransferase 8 (UDP-galactoseceramide galactosyltransferase) | 208358_s_at | 1.7 | NM_003360.1 |
| THY1 | Thy-1 cell surface antigen | 208850_s_at | 1.7 | AL558479 |
| COL4A6 | Similar to collagen, type IV, alpha 6 | 210945_at | 1.7 | BC005305.1 |
| SLUG | slug (chicken homolog), zinc finger protein | 213139_at | 1.7 | AI572079 |
| PON3 | paraoxonase-3 | 213695_at | 1.7 | L48516.1 |
| EMS1 | mammary tumor and squamous cell carcinoma-associated (p80/85 src substrate) | 214073_at | 1.7 | BG475299 |
| ADAMTS2 | a disintegrin and metalloprotease withthrombospondin motifs-2, isoform 1 | 214454_at | 1.7 | NM_014244.1 |
| HJURP | Holliday junction recognition protein | 218726_at | 1.7 | NM_018410.1 |
| SLC12AB | solute carrier family 12), member 8 | 219874_at | 1.7 | NM_024628.1 |
| ST7 | potential tumor suppressor | 220254_at | 1.7 | NM_013437.1 |
| MAGE1 | Melanoma antigen 1 | 221261_x_at | 1.7 | NM_030801.1 |
| NY-BR-81 | protein phosphatase 1, regulatory (inhibitor) subunit 14C | 226907_at | 1.7 | N32557 |
| COL12A1 | collagen type XII alpha-1 | 231766_s_at | 1.7 | U73778.1 |
| NTF5 | neurotrophin 5 precursor | 231785_at | 1.7 | NM_006179.1 |
| NICE-2 | S100 calcium binding protein A7A | 232170_s_at | 1.7 | AJ243672.2 |
| PNKD | paroxysmal nonkinesigenic dyskinesia | 233177_s_at | 1.7 | AB033010.1 |
| FANCB | Fanconi anemia, complementation group B | 1553244_a | 1.7 | NM_152633.1 |
| ANO1 | anoctamin 1, calcium activated chloride channel | 1555269_a_at | 1.7 | BC033036.1 |
| TROAP | trophinin associated protein (tastin) | 1568596_a_at | 1.7 | AI199355 |
| BIRC5 | baculoviral IAP repeat-containing protein 5, survivin | 202095_s_at | 1.6 | NM_001168.1 |
| SLC16A1 | solute carrier family 16 (monocarboxylic acid transporters), member 1 | 202234_s_at | 1.6 | BF511091 |
| FADD | Fas (TNFRSF6)-associated via death domain | 202535_at | 1.6 | NM_003824.1 |
| CDH3 | cadherin 3, type 1, P-cadherin (placental) | 203256_at | 1.6 | NM_001793.1 |
| NELL2 | nel (chicken)-like 2 | 203413_at | 1.6 | NM_006159.1 |
| DFNA5 | deafness, autosomal dominant 5 protein | 203695_s_at | 1.6 | NM_004403.1 |
| SCG2 | secretogranin II precursor | 204035_at | 1.6 | NM_003469.2 |
| SC65 | synaptonemal complex protein SC65 | 204078_at | 1.6 | NM_006455.1 |
| STK15 | serinethreonine kinase 15 | 204092_s_at | 1.6 | NM_003600.1 |
| CSPG2 | chondroitin sulfate proteoglycan 2 (versican) | 204619_s_at | 1.6 | BF590263 |
| CPB1 | pancreatic carboxypeptidase B1 precursor | 205509_at | 1.6 | NM_001871.1 |
| SLC6A15 | solute carrier family 6 ), member 15 | 206376_at | 1.6 | NM_018057.1 |
| AIM2 | absent in melanoma 2 | 206513_at | 1.6 | NM_004833.1 |
| SCYA7 | monocyte chemotactic protein 3 precursor | 208075_s_at | 1.6 | NM_006273.2 |
| GPC3 | glypican | 209220_at | 1.6 | L47125.1 |
| RGSZ1 | regulator of G protein signaling | 210138_at | 1.6 | AF074979.1 |
| COL4A1 | collagen, type IV, alpha 1 | 211981_at | 1.6 | NM_001845.1 |
| MKI67 | antigen identified by monoclonal antibody Ki-67 | 212023_s_at | 1.6 | AU147044 |
| DKC1 | dyskeratosis congenita 1, dyskerin | 216212_s_at | 1.6 | AJ010395 |
| AND-1 | Acidic nucleoplasmic DNA-binding protein 1 | 216228_s_at | 1.6 | AK001538.1 |
| CLDN1 | claudin 1 | 218182_s_at | 1.6 | NM_021101.1 |
| TASP | testis-specific adriamycin sensitivity protein | 218219_s_at | 1.6 | NM_018697.1 |
| RAB6KIFL | RAB6 interacting, kinesin -like (rabkinesin6) | 218755_at | 1.6 | NM_005733.1 |
| USP18 | ubiquitin specific protease 18 | 219211_at | 1.6 | NM_017414.1 |
| [NUDT11](http://www.ncbi.nlm.nih.gov/gene/55190) | nudix (diphosphate linked moiety X)-type motif 11 | 219855_at | 1.6 | NM_018159.1 |
| ITGA11 | integrin alpha-11 subunit precursor | 222899_at | 1.6 | AF109681.1 |
| RAB3IP | RAB3A interacting protein | 223471_at | 1.6 | BC002556.1 |
| PTGFRN | prostaglandin F2 receptor negative regulator | 224950_at | 1.6 | BF476250 |
| ICBP90 | transcription factor | 225655_at | 1.6 | AK025578.1 |
| OS4 | conserved gene amplified in osteosarcoma | 227609_at | 1.6 | AA633203 |
| MTHFD1L | methylenetetrahydrofolate dehydrogenase (NADP+ dependent) 1-like | 231094_s_at | 1.6 | AL035086 |
| ATP6V0D2 | vacuolar H+ ATPase d2 subunit | 1553151_at | 1.6 | AY079172.1 |
| MCTP2 | multiple C2 domains, transmembrane 2 | 1554833_at | 1.6 | BC025708.1 |
| CCDC75 | coiled-coil domain containing 75 | 1559893_at | 1.6 | AK095667.1 |
| FLJ90492 | hypothetical protein FLJ90492 | 1560017_at | 1.6 | AK074973.1 |
| PIP5K2B | phosphatidylinositol-4-phosphate 5-kinase, typeII, beta | 201081_s_at | 1.5 | NM_003559.1 |
| HXB | hexabrachion (tenascin C, cytotactin) | 201645_at | 1.5 | NM_002160.1 |
| MEST | mesoderm specific transcript (mouse) homolog | 202016_at | 1.5 | NM_002402.1 |
| HK2 | hexokinase 2 | 202934_at | 1.5 | AI761561 |
| THBS2 | thrombospondin 2 | 203083_at | 1.5 | NM_003247.1 |
| CDC6 | cell division cycle 6, (S. cerevisiaehomolog) | 203968_s_at | 1.5 | NM_001254.1 |
| TRIP13 | thyroid hormone receptor interactor 13 | 204033_at | 1.5 | NM_004237.1 |
| EDNRA | endothelin receptor type A | 204463_s_at | 1.5 | AU118882 |
| ECGF1 | endothelial cell growth factor 1 (platelet-derived) | 204858_s_at | 1.5 | NM_001953.2 |
| ITGB4 | integrin, beta 4 | 204989_s_at | 1.5 | BF305661 |
| RAGE | renal tumor antigen | 205130_at | 1.5 | NM_014226.1 |
| TC21 | oncogene TC21 | 208456_s_at | 1.5 | NM_012250.1 |
| STK5 | aurora kinase B | 209464_at | 1.5 | AB011446.1 |
| APOL1 | apolipoprotein L-I | 209546_s_at | 1.5 | AF323540.1 |
| BUB1 | putative mitotic checkpoint kinase | 209642_at | 1.5 | AF043294.2 |
| HOXA9 | homeo box A9 | 209905_at | 1.5 | AI246769 |
| hxCT | Cystine glutamate exchanger | 209921_at | 1.5 | AB040875.1 |
| CTSU | cathepsin U | 210074_at | 1.5 | AF070448.1 |
| FZD2 | frizzled (Drosophila) homolog 2 | 210220_at | 1.5 | L37882.1 |
| CNTN1 | h-contactin 2 precursor | 211203_s_at | 1.5 | U07820.1 |
| APOE | apolipoprotein E | 212883_at | 1.5 | AI358867 |
| DXS1357E | accessory proteins BAP31 | 213843_x_at | 1.5 | AW276522 |
| BCAT1 | branched chain aminotransferase 1, cytosolic | 214390_s_at | 1.5 | AI652662 |
| PCDHGC3 | protocadherin 43 | 217426_at | 1.5 | L11372.1 |
| OAS3 | 2-5oligoadenylate synthetase 3 | 218400_at | 1.5 | NM_006187.1 |
| MCM10 | minichromosome maintenance complex component 10 | 220651_s_at | 1.5 | NM_018518.1 |
| CDCA3 | cell division cycle associated 3 | 221436_s_at | 1.5 | NM_031299.1 |
| EGLN3 | egl nine homolog 3 | 222847_s_at | 1.5 | AI378406 |
| HsMCM10 | MCM10 homolog | 222962_s_at | 1.5 | AB042719.1 |
| DMRT2 | DMRT2 terra-like protein | 223704_s_at | 1.5 | AF284225.1 |
| DEFB3 | beta-defensin 3 | 224239_at | 1.5 | AF301470.1 |
| TNFRSF18 | TNF receptor superfamily activation-inducible protein | 224553_s_at | 1.5 | AF117297.1 |
| RAD51 | RAD51 (S. cerevisiae) homolog (E coli RecA homolog) | 224753_at | 1.5 | BE614410 |
| PRG5 | p53-responsive gene 5 | 242204_at | 1.5 | AI242082 |
| MPP4/FL | membrane protein, palmitoylated 4 (MAGUK p55subfamily member 4) | 1552489_s_at | 1.5 | NM_033066.1 |
| CALN1 | calneuron 1 | 1555168_a_at | 1.5 | BC020200.1 |
