# Supplementary material for: Genome-Wide Gene Expression Profile Analyses Identify CTTN as a Potential Prognostic Marker in Esophageal Cancer
Source: PLoS One. 2014 Feb 14;9(2):e88918. doi: 10.1371/journal.pone.0088918 (PMC3925182; doi:10.1371/journal.pone.0088918)
Supplement: Table S2 — Down-regulated genes (≥1.5-fold) in ten cases of esophageal squamous cell carcinoma. (DOC) [file pone.0088918.s003.doc]

Table S2. Down-regulated genes (≥1.5-fold) in ten cases of esophageal squamous cell carcinoma

| **Gene symbol** | **Gene name** | **Probe set ID** | **Fold-**  **change** | **GenBank no.** |
| --- | --- | --- | --- | --- |
| LGI1 | leucine-rich, glioma inactivated 1 | 206349_at | -7.1 | NM_005097.1 |
| GYS2 | glycogen synthase 2 (liver) | 214621_at | -6.2 | S70004.1 |
| MAL | mal, T-cell differentiation protein | 204777_s_at | -5.7 | NM_002371.2 |
| MGB1 | mammaglobin 1 | 206378_at | -5.4 | NM_002411.1 |
| FLG | filaggrin | 215704_at | -5.3 | AL356504 |
| C1ORF10 | chromosome 1 open reading frame 10 | 220090_at | -5.2 | NM_016190.1 |
| TMPRSS11B | transmembrane protease, serine 11B | 1560712_at | -5.2 | AL833167.1 |
| CNGA3 | cyclic nucleotide gated channel alpha 3 | 207261_at | -5 | NM_001298.1 |
| MAB21L2 | MAB21L2 protein | 210302_s_at | -5 | AF262032.1 |
| TFF3 | trefoil factor 3 (intestinal) | 204623_at | -4.9 | NM_003226.1 |
| SGP28 | specific granule protein (28 kDa); cysteine-rich secretory protein-3 | 207802_at | -4.9 | NM_006061.1 |
| TFAP2B | transcription factor AP-2 beta (activating enhancer- binding protein 2 beta) | 214451_at | -4.9 | NM_003221.1 |
| LPRP | lacrimal proline rich protein | 204919_at | -4.8 | NM_007244.1 |
| B3GALT2 | UDP-Gal:betaGlcNAc beta 1,3-galactosyltransferase, polypeptide 2 | 210121_at | -4.8 | AF288390.1 |
| ERBB4 | v-erb-a erythroblastic leukemia viral oncogene homolog 4 (avian) | 214053_at | -4.8 | AW772192 |
| KRTAP3.2 | keratin associated protein 3.2 | 233534_at | -4.8 | AJ406932.1 |
| SOX6 | SRY (sex determining region Y)-box 6 | 1563454_at | -4.8 | AL833302.1 |
| TRDN | triadin | 222287_at | -4.7 | AW969675 |
| SYTL3 | synaptotagmin-like 3 | 1561882_at | -4.6 | BC042966.1 |
| STATH | statherin | 206835_at | -4.5 | NM_003154.1 |
| TACR1 | tachykinin receptor 1 | 208049_s_at | -4.5 | NM_015727.1 |
| MUC5B | mucin 5B, oligomeric mucus/gel-forming | 213432_at | -4.4 | AI697108 |
| GP2 | glycoprotein 2 (zymogen granule membrane) | 206681_x_at | -4.3 | NM_001502.1 |
| DRD1 | dopamine receptor D1 | 214652_at | -4.3 | X58987.1 |
| ABCF2 | ATP-binding cassette, sub-family F (GCN20), member 2 | 207623_at | -4.2 | NM_005692.2 |
| CD163 | CD163 molecule | 216233_at | -4.2 | Z22970.1 |
| K5B | keratin 5b | 1553213_a_at | -4.1 | NM_173352.1 |
| SPINK5 | serine protease inhibitor, Kazal type, 5 | 205185_at | -4 | NM_006846.1 |
| GRAP2 | GRB2-related adaptor protein 2 | 208406_s_at | -4 | NM_004810.1 |
| CD200R | cell surface glycoprotein receptor CD200 | 1552875_a_at | -4 | NM_138939.1 |
| PRSS27 | protease, serine 27 | 232074_at | -3.9 | AW170323 |
| MSMB | microseminoprotein, beta- | 210297_s_at | -3.8 | U22178.1 |
| ARS | ARS component B | 214536_at | -3.8 | NM_020427.1 |
| UPK1A | uroplakin 1A | 214624_at | -3.8 | AA548647 |
| CLCA4 | chloride channel, calcium activated, family member4 | 220026_at | -3.8 | NM_012128.2 |
| ADAM33 | ADAM metallopeptidase domain 33 | 232570_s_at | -3.8 | AL356755 |
| SLC44A4 | solute carrier family 44, member 4 | 1555203_s_at | -3.8 | AF466766.1 |
| PSCA | prostate stem cell antigen | 205319_at | -3.7 | NM_005672.1 |
| LPHB | lipophilin B (uteroglobin family member), prostatein-like | 206799_at | -3.7 | NM_006551.2 |
| EDN3 | endothelin 3 | 208399_s_at | -3.7 | NM_000114.1 |
| ADH1B | alcohol dehydrogenase IB (class I), beta polypeptide | 209612_s_at | -3.7 | M24317.1 |
| B3GALT2 | UDP-Gal:betaGlcNAc beta 1,3-galactosyltransferase, polypeptide 2 | 217452_s_at | -3.7 | Y15014.1 |
| SLC14A1 | Solute carrier family 14, member 1 (Kidd blood group) | 229151_at | -3.7 | BE673587 |
| ADH1B | alcohol dehydrogenase IB (class I), beta polypeptide | 209613_s_at | -3.6 | M21692.1 |
| EPM2A | epilepsy, progressive myoclonus type 2A, Lafora disease (laforin) | 216079_at | -3.6 | AK022721.1 |
| MSMB | microseminoprotein, beta- | 207430_s_at | -3.5 | NM_002443.1 |
| ADH1A | alcohol dehydrogenase 1A (class I), alpha polypeptide | 209614_at | -3.5 | AF153821.1 |
| PLGLB2 | plasminogen-like B2 | 1558603_at | -3.5 | AV688060 |
| SYTL3 | synaptotagmin-like 3 | 1562255_at | -3.5 | AL833750.1 |
| PRH1 | proline-rich protein HaeIII subfamily 1 | 205272_s_at | -3.4 | NM_006250.1 |
| SCEL | sciellin | 206884_s_at | -3.4 | NM_003843.1 |
| KRTHA3A | keratin, hair, acidic,3A | 208483_x_at | -3.4 | NM_004138.1 |
| TKTL1 | transketolase-like 1 | 214183_s_at | -3.4 | X91817.1 |
| PCP4 | Purkinje cell protein 4 | 205549_at | -3.3 | NM_006198.1 |
| TGM3 | transglutaminase 3 (E polypeptide, protein-glutamine-gamma-glutamyltransferase) | 206004_at | -3.3 | NM_003245.1 |
| AZGP1 | alpha-2-glycoprotein 1, zinc-binding | 209309_at | -3.3 | D90427.1 |
| SPINK7 | serine peptidase inhibitor, Kazal type 7 (putative) | 223720_at | -3.3 | AF268198.1 |
| K5B | keratin 5b | 1553212_at | -3.3 | NM_173352.1 |
| DEFB104 | defensin, beta 104 | 1553521_at | -3.3 | NM_080389.1 |
| PPL | periplakin | 203407_at | -3.2 | NM_002705.1 |
| TM4SF3 | transmembrane 4 superfamily member 3 | 203824_at | -3.2 | NM_004616.1 |
| TCN1 | transcobalamin I (vitamin B12 binding protein, R binder family) | 205513_at | -3.2 | NM_001062.1 |
| MGB2 | mammaglobin 2 | 205979_at | -3.2 | NM_002407.1 |
| ECM1 | extracellular matrix protein 1 | 209365_s_at | -3.2 | U65932.1 |
| TJP3 | tight junction protein 3 (zona occludens 3) | 213412_at | -3.2 | NM_014428.1 |
| LOC51190 | neutral sphingomyelinase | 221405_at | -3.2 | NM_016317.1 |
| SCEL | sciellin | 232056_at | -3.2 | AW470178 |
| NKX3-1 | NK3 transcription factor related, locus 1 (Drosophila) | 209706_at | -3.1 | AF247704.1 |
| AQP5 | aquaporin 5 | 213611_at | -3.1 | BF726531 |
| RHCG | Rh type C glycoprotein | 219554_at | -3.1 | NM_016321.1 |
| CNFN | cornifelin | 224329_s_at | -31 | AB049591.1 |
| TFE3 | Transcription factor binding to IGHM enhancer 3 | 1567704_at | -3.1 | AY034077.1 |
| PPP1R3C | protein phosphatase 1, regulatory (inhibitor) subunit 3C | 204284_at | -3 | N26005 |
| TREH | trehalase (brush-border membrane glycoprotein) | 207378_at | -3 | NM_007180.1 |
| CSF3 | colony stimulating factor 3 (granulocyte) | 207442_at | -3 | NM_000759.1 |
| KRT13 | keratin 13 | 207935_s_at | -3 | NM_002274.1 |
| CEACAM1 | carcinoembryonic antigen-related cell adhesion molecule 1 (biliary glycoprotein) | 209498_at | -3 | X16354.1 |
| CYP4B1 | cytochrome P450, family 4, subfamily B, polypeptide 1 | 210096_at | -3 | J02871.1 |
| KIR2DL5.3 | killer cell Ig-like receptor KIR2DL5.3 | 211410_x_at | -3 | AF217487.1 |
| TRHDE | thyrotropin-releasing hormone degrading ectoenzyme | 219937_at | -3 | NM_013381.1 |
| DCT | dopachrome tautomerase (dopachrome delta- isomerase, tyrosine-related protein 2) | 205337_at | -2.9 | AL139318 |
| MMRN | multimerin | 205612_at | -2.9 | NM_007351.1 |
| CGM2 | carcinoembryonic antigen | 206198_s_at | -2.9 | L31792.1 |
| CEACAM7 | carcinoembryonic antigen-related cell adhesion molecule 7 | 206199_at | -2.9 | NM_006890.1 |
| CEACAM1 | carcinoembryonic antigen-related cell adhesion molecule 1 (biliary glycoprotein) | 206576_s_at | -2.9 | NM_001712.1 |
| FCT3A | alpha-1,3 fucosyltransferase 6 | 210398_x_at | -2.9 | M98825.1 |
| PROML1 | prominin (mouse)-like 1 | 204304_s_at | -2.8 | NM_006017.1 |
| FOLR1 | folate receptor 1 (adult) | 204437_s_at | -2.8 | NM_016725.1 |
| TMPRSS2 | transmembrane protease, serine 2 | 205102_at | -2.8 | NM_005656.1 |
| PDGH | NAD+-dependent 15-hydroxyprostaglandin dehydrogenase | 211548_s_at | -2.8 | J05594.1 |
| HPGD | hydroxyprostaglandin dehydrogenase 15-(NAD) | 211549_s_at | -2.8 | U63296.1 |
| TMPRSS2 | androgen-regulated serine protease TMPRSS2 precursor | 211689_s_at | -2.8 | AF270487.1 |
| ANXA9 | annexin A9 | 211712_s_at | -2.8 | BC005830.1 |
| FMO2 | flavin containing monooxygenase 2 (non-functional) | 211726_s_at | -2.8 | BC005894.1 |
| CEACAM1 | carcinoembryonic antigen-related cell adhesion molecule 1 (biliary glycoprotein) | 211889_x_at | -2.8 | D12502.1 |
| CLIC3 | chloride intracellular channel 3 | 219529_at | -2.8 | NM_004669.1 |
| DESC1 | DESC1 protein | 220431_at | -2.8 | NM_014058.1 |
| AK155 | AK155 protein | 221111_at | -2.8 | NM_018402.1 |
| SLC5A7 | solute carrier family 5 (choline transporter), member 7 | 222967_at | -2.8 | AB043997.1 |
| SORBS2 | sorbin and SH3 domain containing 2 | 225728_at | -2.8 | AI659533 |
| GBP6 | Guanylate binding protein family, member 6 | 1559606_at | -2.8 | AL703282 |
| HPGD | hydroxyprostaglandin dehydrogenase 15-(NAD) | 203913_s_at | -2.7 | AL574184 |
| CYP11A | cytochrome P450, subfamily XIA (cholesterol side chain cleavage) | 204309_at | -2.7 | NM_000781.1 |
| SCYA14 | small inducible cytokine subfamily A (Cys-Cys), member 14 | 205392_s_at | -2.7 | NM_004166.1 |
| P11 | protease, serine, 22 | 206605_at | -2.7 | NM_006025.1 |
| FCER1A | Fc fragment of IgE, high affinity I, receptor for; alpha polypeptide | 211734_s_at | -2.7 | BC005912.1 |
| DPT | dermatopontin | 213068_at | -2.7 | AI146848 |
| TF | transferrin | 214063_s_at | -2.7 | AI073407 |
| NICE-1 | NICE-1 protein | 220620_at | -2.7 | NM_019060.1 |
| ECRG4 | esophageal cancer related gene 4 protein | 223623_at | -2.7 | AF325503.1 |
| GBP6 | Guanylate binding protein family, member 6 | 1559607_s_at | -2.7 | AL703282 |
| HPGD | hydroxyprostaglandin dehydrogenase 15-(NAD) | 203914_x_at | -2.6 | NM_000860.1 |
| KRTHA2 | keratin, hair, acidic,2 | 207146_at | -2.6 | NM_002278.1 |
| ALOX12 | arachidonate 12-lipoxygenase | 207206_s_at | -2.6 | NM_000697.1 |
| HRBL | HIV-1 Rev binding protein-like | 222362_at | -2.6 | H07885 |
| EHF | ETS-family transcription factor EHF | 222932_at | -2.6 | AF203977.1 |
| TMEFF2 | transmembrane protein with EGF-like and two follistatin-like domains 2 | 223557_s_at | -2.6 | AB017269.1 |
| SYCP3 | synaptonemal complex protein 3 | 1553599_a_at | -2.6 | AF492003.1 |
| TNA | tetranectin (plasminogen-binding protein) | 205200_at | -2.5 | NM_003278.1 |
| KRT4 | keratin 4 | 213240_s_at | -2.5 | X07695.1 |
| KRT19P2 | keratin 19 pseudogene 2 | 217350_at | -2.5 | AB041269.1 |
| ROPN1B | ropporin, rhophilin associated protein 1B | 220425_x_at | -2.5 | NM_017578.1 |
| MMP27 | matrix metalloprotease 27 | 220783_at | -2.5 | NM_022122.1 |
| PDK4 | pyruvate dehydrogenase kinase, isozyme 4 | 225207_at | -2.5 | AV707102 |
| CTXL | cortic al thymocyte receptor (X. laevis CTX) like | 228232_s_at | -2.5 | NM_014312.1 |
| NPAS3 | Neuronal PAS domain protein 3 | 230412_at | -2.5 | BF196935 |
| LYNX1 | Ly6/neurotoxin 1 | 1554179_s_at | -2.5 | BC032306.1 |
| IGSF10 | immunoglobulin superfamily, member 10 | 1556579_s_at | -2.5 | AF087980.1 |
| DF | D component of complement (adipsin) | 205382_s_at | -2.4 | NM_001928.1 |
| NPY1R | neuropeptide Y receptor Y1 | 205440_s_at | -2.4 | NM_000909.1 |
| ASPA | aspartoacylase (aminoacylase 2, Canavan disease) | 206030_at | -2.4 | NM_000049.1 |
| PTGDS | prostaglandin D2 synthase 21kDa (brain) | 211663_x_at | -2.4 | M61900.1 |
| TNXB | tenascin XB | 216333_x_at | -2.4 | M25813.1 |
| ARGBP2 | ArgAbl-interacting protein ArgBP2 | 204288_s_at | -2.3 | NM_021069.1 |
| GABRP | gamma-aminobutyric acid (GABA) A receptor, pi | 205044_at | -2.3 | NM_014211.1 |
| LCN1 | lipocalin 1 (protein migrating faster than albumin, tear prealbumin) | 207930_at | -2.3 | NM_002297.1 |
| DPT | dermatopontin | 207977_s_at | -2.3 | NM_001937.2 |
| FAM107A | family with sequence similarity 107, member A | 209074_s_at | -2.3 | AL050264.1 |
| P2RX1 | purinergic receptor P2X, ligand-gated ion channel, 1 | 210401_at | -2.3 | U45448.1 |
| TTY9 | testis transcript Y 9 | 211460_at | -2.3 | AF332238.1 |
| BGPa | alternatively spliced biliary glycoprotein | 211883_x_at | -2.3 | M76742.1 |
| FUT6 | alpha (1,3) fucosyltransferase | 211885_x_at | -2.3 | U27332.1 |
| PTGDS | prostaglandin D2 synthase (21kD, brain) | 212187_x_at | -2.3 | NM_000954.1 |
| IL1RN | interleukin 1 receptor antagonist | 212659_s_at | -2.3 | AW083357 |
| LDB3 | LIM domain binding 3 | 213371_at | -2.3 | AI803302 |
| RLN2 | relaxin 2 (H2) | 214519_s_at | -2.3 | NM_005059.1 |
| TOX3 | TOX high mobility group box family member 3 | 214774_x_at | -2.3 | AK027006.1 |
| SERPINB13 | serpin peptidase inhibitor, clade B (ovalbumin), member 13 | 216258_s_at | -2.3 | BE148534 |
| EPB41L4 | erythrocyte protein band 4.1-like 4 | 220120_s_at | -2.3 | NM_022140.1 |
| ANGPTL1 | angiopoietin-like 1 | 231773_at | -2.3 | BF002046 |
| SCIN | scinderin | 1552365_at | -2.3 | NM_033128.1 |
| RPS15 | Ribosomal protein S15 | 1563014_at | -2.3 | BC006431.1 |
| SFRP1 | secreted frizzled-related protein 1 | 202037_s_at | -2.2 | NM_003012.2 |
| HMGCS2 | 3-hydroxy-3-methylglutaryl-Coenzyme A synthase 2 (mitochondrial) | 204607_at | -2.2 | NM_005518.1 |
| SERPINB2 | serine (or cysteine) proteinase inhibitor, clade B (ovalbumin), member 2 | 204614_at | -2.2 | NM_002575.1 |
| BBOX1 | butyrobetaine (gamma), 2-oxoglutarate dioxygenase (gamma-butyrobetaine hydroxylase) 1 | 205363_at | -2.2 | NM_003986.1 |
| NMU | neuromedin U | 206023_at | -2.2 | NM_006681.1 |
| NELL1 | nel (chicken)-like 1 | 206089_at | -2.2 | NM_006157.1 |
| TNXA | tenascin XA | 206093_x_at | -2.2 | NM_007116.1 |
| BMX | BMX non-receptor tyrosine kinase | 206464_at | -2.2 | NM_001721.1 |
| P2RY14 | purinergic receptor P2Y, G-protein coupled, 14 | 206637_at | -2.2 | NM_014879.1 |
| FLT3 | fms-related tyrosine kinase 3 | 206674_at | -2.2 | NM_004119.1 |
| EPHX2 | clone 129-13 soluble epoxide hydrolase | 209368_at | -2.2 | AF233336.1 |
| CYP2E1 | cytochrome P450-2E1 | 209975_at | -2.2 | AF182276.1 |
| ANXA9 | annexin A9 | 210085_s_at | -2.2 | AF230929.1 |
| PTGDS | prostaglandin D2 synthase 21kDa (brain) | 211748_x_at | -2.2 | BC005939.1 |
| KLRB1 | killer cell lectin-like receptor subfamily B, member 1 | 214470_at | -2.2 | NM_002258.1 |
| VLCS-H1 | VLCS-H1 protein | 219932_at | -2.2 | NM_014031.1 |
| IL1F6 | interleukin 1 family, member 6 (epsilon) | 221404_at | -2.2 | NM_014440.1 |
| SDPR | serum deprivation response | 222717_at | -2.2 | BF982174 |
| ADRB1 | adrenergic, beta-1-, receptor | 229309_at | -2.2 | AI625747 |
| ATP6V1C2 | ATPase, H+ transporting, lysosomal 42kDa, V1 subunit C isoform 2 | 1552532_a_at | -2.2 | NM_144583.1 |
| RSPO2 | R-spondin 2 homolog (Xenopus laevis) | 1554012_at | -2.2 | BC027938.1 |
| ZNF527 | zinc finger protein 527 | 1570238_at | -2.2 | BC014325.1 |
| ATP1A2 | ATPase, Na+K+ transporting, alpha 2 (+) polypeptide | 203296_s_at | -2.1 | NM_000702.1 |
| KIAA0273 | KIAA0273 gene product | 205325_at | -2.1 | NM_014759.1 |
| CLDN10 | claudin 10 | 205328_at | -2.1 | NM_006984.1 |
| SCNN1B | sodium channel, nonvoltage-gated 1, beta (Liddle syndrome) | 205464_at | -2.1 | NM_000336.1 |
| DNASE1L3 | deoxyribonuclease I-like 3 | 205554_s_at | -2.1 | NM_004944.1 |
| TGM1 | transglutaminase 1 (K polypeptide epidermal type I, protein-glutamine-gamma-glutamyltransferase) | 206008_at | -2.1 | NM_000359.1 |
| PIP | prolactin-induced protein | 206509_at | -2.1 | NM_002652.1 |
| CH25H | cholesterol 25-hydroxylase | 206932_at | -2.1 | NM_003956.1 |
| PRSS3 | protease, serine, 3 (trypsin 3) | 207463_x_at | -2.1 | NM_002771.1 |
| CYP2C18 | cytochrome P450, subfamily IIC (mephenytoin 4-hydroxylase), polypeptide 18 | 208126_s_at | -2.1 | NM_000772.1 |
| FY | Duffy blood group | 208335_s_at | -2.1 | NM_002036.1 |
| GP2 | pancreatic zymogen granule membrane associated protein GP2 beta form | 209687_at | -2.1 | NM_016295.1 |
| SCN3A | voltage-gated sodium channel alpha subunit splice variant SCN3A-s | 210432_s_at | -2.1 | AF225986.1 |
| SERPINB13 | serpin peptidase inhibitor, clade B (ovalbumin), member 13 | 211362_s_at | -2.1 | AF169949.1 |
| CGM2 | carcinoembryonic antigen 2b | 211848_s_at | -2.1 | AF006623.1 |
| PRSS3 | protease, serine, 3 (mesotrypsin) | 213421_x_at | -2.1 | AW007273 |
| ITGA8 | integrin, alpha 8 | 214265_at | -2.1 | AI193623 |
| CD1E | CD1e molecule | 215784_at | -2.1 | AA309511 |
| DAZ4 | deleted in azoospermia 4 | 216351_x_at | -2.1 | AF248483.1 |
| OGN | osteoglycin | 222722_at | -2.1 | AV700059 |
| LETAL | lymphocyte effector toxicity activation ligand | 1552777_a_at | -2.1 | NM_139165.1 |
| EMP1 | epithelial membrane protein 1 | 201325_s_at | -2 | NM_001423.1 |
| SARP2 | secreted apoptosis related protein 2 | 202036_s_at | -2 | AF017987.1 |
| CRABP2 | cellular retinoic acid-binding protein 2 | 202575_at | -2 | NM_001878.2 |
| PLCB4 | phospholipase C, beta 4 | 203895_at | -2 | AL535113 |
| ABCA8 | ATP-binding cassette, sub-family A (ABC1), member 8 | 204719_at | -2 | NM_007168.1 |
| TGFBR3 | transforming growth factor, beta receptor III (betaglycan, 300kD) | 204731_at | -2 | NM_003243.1 |
| NR3C2 | nuclear receptor subfamily 3, group C, member 2 | 205259_at | -2 | NM_000901.1 |
| FXYD1 | FXYD domain-containing ion transport regulator 1 (phospholemman) | 205384_at | -2 | NM_005031.2 |
| SULT2B1 | sulfotransferase family, cytosolic, 2B, member 1 | 205759_s_at | -2 | NM_004605.1 |
| NLGN1 | neuroligin 1 | 205893_at | -2 | NM_014932.1 |
| PDK4 | pyruvate dehydrogenase kinase, isoenzyme 4 | 205960_at | -2 | NM_002612.1 |
| PYR3 | ryanodine receptor 3 | 206306_at | -2 | NM_001036.1 |
| MAD | MAX dimerization protein | 206877_at | -2 | NM_002357.1 |
| SH2D1A | SH2 domain protein 1A isoform C | 211209_x_at | -2 | AF100540.1 |
| DIO2 | deiodinase, iodothyronine, type II | 211215_x_at | -2 | AB041843.1 |
| FUT6 | alpha (1,3) fucosyltransferase | 211465_x_at | -2 | U27335.1 |
| SERPINB4 | serpin peptidase inhibitor, clade B (ovalbumin), member 4 | 211906_s_at | -2 | AB046400.1 |
| ZFP36L1 | Zinc finger protein 36, C3H type-like 1 | 213284_at | -2 | BG482928 |
| OGN | osteoglycin (osteoinductive factor, mimecan) | 218730_s_at | -2 | NM_014057.1 |
| PCOLCE2 | procollagen C-endopeptidase enhancer 2 | 219295_s_at | -2 | NM_013363.1 |
| DUOX1 | dual oxidase 1 | 219597_s_at | -2 | NM_017434.1 |
| EHF | Ets homologous factor | 219850_s_at | -2 | NM_012153.1 |
| CYP2C9 | cytochrome P450, subfamily IIC (mephenytoin 4-hydroxylase), polypeptide 9 | 220017_x_at | -2 | NM_000771.2 |
| NBEA | neurobeachin | 221207_s_at | -2 | NM_015678.1 |
| CIDEA | cell death-inducing DFFA-like effector a | 221295_at | -2 | NM_001279.1 |
| CLDN17 | claudin 17 | 221328_at | -2 | NM_012131.1 |
| IL22 | interleukin 22 | 222974_at | -2 | AF279437.1 |
| ANGPTL1 | angiopoietin-like 1 | 224339_s_at | -2 | AB056476.1 |
| ST6GALNAC1 | ST6(alpha-N-acetyl-neuraminyl-2,3-beta-galactosyl-1,3)-N-acetylgalactosaminide alpha-2,6-sialyltransferase 1 | 227725_at | -2 | Y11339.2 |
| ZIK1 | zinc finger protein interacting with K protein 1 homolog (mouse) | 232774_x_at | -2 | AC003682 |
| CYPIIE1 | cytochrome P450IIE1 (ethanol-inducible) gene | 1431_at | -1.9 | J02843 |
| C7 | complement component 7 (C7) | 202992_at | -1.9 | NM_000587.1 |
| AQP3 | aquaporin 3 | 203747_at | -1.9 | NM_004925.2 |
| CEACAM6 | Carcinoembryonic antigen-related cell adhesion molecule 6 (non-specific cross reacting antigen) | 203757_s_at | -1.9 | BC005008.1 |
| ACPP | acid phosphatase, prostate (ACPP) | 204393_s_at | -1.9 | NM_001099.2 |
| KIT | v-kit Hardy-Zuckerman 4 feline sarcoma viral oncogene homolog (KIT) | 205051_s_at | -1.9 | NM_000222.1 |
| KLK13 | kallikrein 13 | 205783_at | -1.9 | NM_015596.1 |
| ZNF145 | zinc finger protein 145 (Kruppel-like, expressed in promyelocytic leukemia) | 205883_at | -1.9 | NM_006006.1 |
| HML2 | macrophage lectin 2 (calcium dependent) | 206682_at | -1.9 | NM_006344.1 |
| RALDH2 | retinaldehyde dehydrogenase 2 | 207016_s_at | -1.9 | NM_003888.1 |
| HBNF-1 | Human nerve growth factor | 209466_x_at | -1.9 | M57399.1 |
| Bft | hindlimb expressed homeobox protein backfoot | 209587_at | -1.9 | U70370.1 |
| CD24 | Cell surface antigen | 209772_s_at | -1.9 | X69397.1 |
| FLJ11129 | leucine-rich repeat neuronal protein 3 | 209840_s_at | -1.9 | AL442092.1 |
| PLP1 | proteolipid protein | 210198_s_at | -1.9 | BC002665.1 |
| SERPINB1 | serine (or cysteine) proteinase inhibitor, cladeB (ovalbumin), member 1 | 212268_at | -1.9 | NM_030666.1 |
| GPX3 | glutathione peroxidase 3 (plasma) | 214091_s_at | -1.9 | AW149846 |
| MBD2 | methyl-CpG binding domain protein 2 | 214397_at | -1.9 | AI827820 |
| IVL | involucrin | 214599_at | -1.9 | NM_005547.1 |
| EPS8L2 | EPS8-like 2 | 218180_s_at | -1.9 | NM_022772.1 |
| RAGD | Rag D | 221524_s_at | -1.9 | AF272036.1 |
| BRDG1 | BCR downstream signaling 1 | 1554343_a_at | -1.9 | BC014958.1 |
| DSCR1L1 | Down syndrome critical region gene 1-like 1 | 203498_at | -1.8 | NM_005822.1 |
| APM2 | adipose specific 2 (APM2) | 203571_s_at | -1.8 | NM_006829.1 |
| EVPL | envoplakin | 204503_at | -1.8 | NM_001988.1 |
| WIF-1 | Wnt inhibitory factor-1 | 204712_at | -1.8 | NM_007191.1 |
| TCF21 | transcription factor 21 (TCF21) | 204931_at | -1.8 | NM_003206.1 |
| PPP1R1A | protein phosphatase 1, regulatory (inhibitor)subunit 1A | 205478_at | -1.8 | NM_006741.1 |
| CYP3A5 | cytochrome P450, subfamily IIIA, polypeptide 5 | 205765_at | -1.8 | NM_000777.1 |
| CD1C | CD1C antigen, c polypeptide | 205987_at | -1.8 | NM_001765.1 |
| IL18 | interleukin 18 (interferon-gamma-inducing factor) | 206295_at | -1.8 | NM_001562.1 |
| IL8RB | interleukin 8 receptor, beta | 207008_at | -1.8 | NM_001557.1 |
| H963 | platelet activating receptor homolog | 207651_at | -1.8 | NM_013308.1 |
| MUC1 | mucin 1, transmembrane | 207847_s_at | -1.8 | NM_002456.1 |
| SCN6A | sodium channel, voltage-gated, type VI, alpha polypeptide | 207864_at | -1.8 | NM_002976.1 |
| AGR2 | putative secreted protein XAG | 209173_at | -1.8 | AF088867.1 |
| DVS27 | DVS27-related protein | 209821_at | -1.8 | AB024518.1 |
| CCNG2 | cyclin G2 | 211559_s_at | -1.8 | L49506.1 |
| LPIN1 | lipin 1 | 212274_at | -1.8 | AV705559 |
| MFAP4 | microfibrillar-associated protein 4 | 212713_at | -1.8 | R72286 |
| CLU | clusterin | 222043_at | -1.8 | AI982754 |
| ADAMTS1 | similar to Homo sapiens metalloproteinase with thrombospondin type 1 motifs | 222162_s_at | -1.8 | AK023795.1 |
| Itln | intelectin | 223597_at | -1.8 | AB036706.1 |
| ST5 | suppression of tumorigenicity 5 | 224839_s_at | -1.8 | BG328998 |
| KIAA1481 | shroom family member 3 | 225548_at | -1.8 | AB040914.1 |
| LOC57822 | grainyhead-like 3 (Drosophila) | 232116_at | -1.8 | AL137763.1 |
| KIAA1758 | cortactin binding protein 2 | 232136_s_at | -1.8 | AB051545.1 |
| SERPINB11 | serine (or cysteine) proteinase inhibitor, clade B (ovalbumin), member 11 | 1552463_at | -1.8 | NM_080475.1 |
| ALOX15B | 15-lipoxygenase 2 splice variant b | 1555416_a_at | -1.8 | AF468053.1 |
| ITM2A | integral membrane protein 2A | 202746_at | -1.7 | AL021786 |
| ZNF185 | zinc finger protein 185 (LIM domain) | 203585_at | -1.7 | NM_007150.1 BRG1 |
| PCAF | p300CBP-associated factor | 203845_at | -1.7 | AV727449 |
| NEBL | nebulette | 203961_at | -1.7 | AL157398 |
| CLDN5 | transmembrane protein claudin 5 | 204482_at | -1.7 | NM_003277.1 |
| HLF | hepatic leukemia factor | 204754_at | -1.7 | W60800 |
| C44A | GPI-anchored metastasis-associated protein homolog | 204952_at | -1.7 | NM_014400.1 |
| RNASE4 | ribonuclease, RNase A family, 4 | 205158_at | -1.7 | NM_002937.1 |
| BAI3 | brain-specific angiogenesis inhibitor 3 | 205638_at | -1.7 | NM_001704.1 |
| CTSG | cathepsin G | 205653_at | -1.7 | NM_001911.1 |
| Ablim3 | actin binding LIM protein family, member 3 | 205730_s_at | -1.7 | NM_014945.1 |
| PTN | pleiotrophin | 209465_x_at | -1.7 | AL565812 |
| GPR37 | putative endothelin receptor type B-likeprotein | 209631_s_at | -1.7 | U87460.1 |
| LOC57803 | chordin-like1 | 209763_at | -1.7 | AL049176 |
| SCYA19 | beta chemokine Exodus-3 | 210072_at | -1.7 | U88321.1 |
| CEACAM4 | non-specific cross reacting antigen | 211657_at | -1.7 | M18728.1 |
| CNR1 | cannabinoid receptor (CNR1) gene | 213436_at | -1.7 | U73304 |
| OR7E14P | olfactory receptor, family 7, subfamily E, member 13 pseudogene | 217551_at | -1.7 | AA719797 |
| MGC10848 | inter-alpha (globulin) inhibitor H5 | 219064_at | -1.7 | NM_030569.1 |
| FLJ20701 | phosphotyrosine interaction domain containing 1 | 219093_at | -1.7 | NM_017933.1 |
| C4orf31 | chromosome 4 open reading frame 31 | 219747_at | -1.7 | NM_024574.1 |
| GNA14 | guanine nucleotide binding protein (G protein),alpha 14 | 220108_at | -1.7 | NM_004297.1 |
| LANGERIN | Langerhans cell specific c-type lectin | 220428_at | -1.7 | NM_015717.1 |
| NMES1 | normal mucosa of esophagus specific 1 | 223484_at | -1.7 | AF228422.1 |
| KIAA1307 | ubiquitin protein ligase E3 component n-recognin 4 | 231889_at | -1.7 | AB037728.1 |
| DKFZp434B0610 | sperm associated antigen 17 | 233516_s_at | -1.7 | AL137581.1 |
| FLJ25179 | alpha-2-macroglobulin-like 1 | 1564307_a_at | -1.7 | AL832750.1 |
| MEGF5 | multiple EGF-like domains protein 5 | 203812_at | -1.6 | AB011538.1 |
| RECK, ST15 | RECK protein precursor | 205407_at | -1.6 | NM_021111.1 |
| HS3ST1 | heparan sulfate D-glucosaminyl3-O-sulfotransferase 1 precursor | 205466_s_at | -1.6 | NM_005114.1 |
| CLGN | calmegin | 205830_at | -1.6 | NM_004362.1 |
| KLRC2 | killer cell lectin-like receptor subfamily C,member 2 | 206785_s_at | -1.6 | NM_002260.2 |
| CASQ2 | cardiac calsequestrin | 207317_s_at | -1.6 | NM_001232.1 |
| MS4A1 | membrane-spanning 4-domains, subfamily A, member1 | 207496_at | -1.6 | NM_000139.1 |
| ABCC9 | ATP-binding cassette, sub-family C, member 9,isoform SUR2A | 208462_s_at | -1.6 | NM_005691.1 |
| PEG3 | paternally expressed 3 | 209242_at | -1.6 | AL042588 |
| ID4 | inhibitor of DNA binding 4 | 209292_at | -1.6 | AL022726 |
| DUSP5 | protein tyrosine phosphatase | 209457_at | -1.6 | U16996.1 |
| GATA2 | GATA-binding protein 2 | 209710_at | -1.6 | AL563460 |
| mdr1 | P-glycoprotein | 209994_s_at | -1.6 | AF016535.1 |
| MYOC | myocilin | 210155_at | -1.6 | D88214.1 |
| RGS13 | regulator of G protein signaling | 210258_at | -1.6 | AF030107.1 |
| KCNAB1 | K+ channel beta 1a subunit | 210471_s_at | -1.6 | U33428.1 |
| SASH1 | SAM and SH3 domain containing 1 | 213236_at | -1.6 | AK025495.1 |
| HF1 | complement factor H 38-kDa N-terminal fragment | 213800_at | -1.6 | X04697.1 |
| ECHDC2 | enoyl CoA hydratase domain containing 2 | 218552_at | -1.6 | NM_018281.1 |
| XLKD1 | extracellular link domain-containing 1 | 219059_s_at | -1.6 | AL574194 |
| NET-2 | tetraspanin 12 | 219274_at | -1.6 | NM_012338.1 |
| WFDC1 | WAP four-disulfide core domain 1 | 219478_at | -1.6 | NM_021197.1 |
| SUT1 | sulfate transporter 1,mRNA. | 219824_at | -1.6 | NM_012450.1 |
| MGC2742 | hypothetical protein MGC2742 | 219856_at | -1.6 | NM_023938.1 |
| HAGE | DEAD-box protein | 220004_at | -1.6 | NM_018665.1 |
| LRRC31 | leucine rich repeat containing 31 | 220622_at | -1.6 | NM_024727.1 |
| KLK12 | kallikrein 12 | 220782_x_at | -1.6 | NM_019598.1 |
| KIAA1324 | KIAA1324 | 221874_at | -1.6 | AB037745.1 |
| CTRP7 | complement-c1q tumor necrosis factor-related protein | 223877_at | -1.6 | AF329839.1 |
| RDH | retinol dehydrogenase homolog isoform-1 (RDH) | 223952_x_at | -1.6 | AF240698.1 |
| CCK1 | CCK1 protein | 224027_at | -1.6 | AF110384.1 |
| CHAK2 | channel kinase 2 | 224412_s_at | -1.6 | AF350881.1 |
| MYBBP1A | MYB binding protein (P160) 1a | 225671_at | -1.6 | AL568674 |
| FLJ12577 | hypothetical protein FLJ12577 | 225915_at | -1.6 | gb:AL138875 |
| OCLN | occludin | 227492_at | -1.6 | AI829721 |
| SNURF | SNRPN upstream reading frame | 228370_at | -1.6 | BF114870 |
| FLJ30296 | patched domain containing 1 | 1552848_a_at | -1.6 | NM_173495.1 |
| FHL-1 | Human heart protein with four and a half LIM domains | 201539_s_at | -1.5 | U29538.1 |
| EVA1 | epithelial V-like antigen 1 | 203779_s_at | -1.5 | NM_005797.1 |
| CP | ceruloplasmin (ferroxidase) | 204846_at | -1.5 | NM_000096.1 |
| FABP7 | fatty acid binding protein 7, brain | 205030_at | -1.5 | NM_001446.1 |
| CPA3 | mast cell carboxypeptidase A3 precursor | 205624_at | -1.5 | NM_001870.1 |
| RETL2 | RET ligand 2 (RETL2) | 205721_at | -1.5 | U97145.1 |
| RELN | reelin | 205923_at | -1.5 | NM_005045.1 |
| PTX3 | pentaxin-related gene, rapidly induced by IL-1 beta (PTX3) | 206157_at | -1.5 | NM_002852.1 |
| NME5 | non-metastatic cells 5, protein expressed in (nucleoside-diphosphate kinase) (NME5) | 206197_at | -1.5 | NM_003551.1 |
| KCNA5 | potassium voltage-gated channel, shaker-related subfamily, member 5 (KCNA5) | 206762_at | -1.5 | NM_002234.1 |
| APM1 | adipose most abundant gene transcript 1 | 207175_at | -1.5 | NM_004797.1 |
| LGALS2 | lectin, galactoside-binding, soluble, 2(galectin 2) | 208450_at | -1.5 | NM_006498.1 |
| ITGA7 | integrin alpha-7 | 209663_s_at | -1.5 | AF072132.1 |
| CES2 | carboxylesterase precursor | 209668_x_at | -1.5 | D50579.1 |
| TCRG | Human T-cell receptor gamma chain VJCI-CII-CIII region mRNA, complete cds. | 209813_x_at | -1.5 | M16768.1 |
| DLGAP2 | discs, large (Drosophila) homolog-associated protein 2 | 210227_at | -1.5 | AF119817.1 |
| MYO6 | myosin VI (MYO6) | 210480_s_at | -1.5 | U90236.2 |
| ADH7 | alcohol dehydrogenase | 210505_at | -1.5 | U07821.1 |
| FUT2 | Similar to fucosyltransferase 2 (secretor statusincluded) | 210608_s_at | -1.5 | BC001899.1 |
| GPD1L | glycerol-3-phosphate dehydrogenase 1-like | 212510_at | -1.5 | AA135522 |
| COL14A1 | collagen, type XIV, alpha 1 (undulin) | 212865_s_at | -1.5 | BF449063 |
| DNAJC8 | DnaJ (Hsp40) homolog, subfamily C, member 8 | 213397_x_at | -1.5 | AI761728 |
| SELENBP1 | selenium binding protein 1 (SELENBP1) | 214433_s_at | -1.5 | NM_003944.1 |
| MLPH | melanophilin | 218211_s_at | -1.5 | NM_024101.1 |
| OBFC1 | oligonucleotide/oligosaccharide-binding fold containing 1 | 219100_at | -1.5 | NM_024928.1 |
| FLJ22622 | RAB11 family interacting protein 1 | 219681_s_at | -1.5 | NM_025151.1 |
| FLJ14084 | transmembrane protein 35 | 219685_at | -1.5 | NM_021637.1 |
| LRRC2 | leucine-rich repeat-containing 2 | 219949_at | -1.5 | NM_024512.1 |
| ACT | activator of CREM in testis (ACT) | 220170_at | -1.5 | NM_020482.1 |
| NSBP1 | nucleosomal binding protein 1 | 221606_s_at | -1.5 | BC005342.1 |
| GALNT12 | UDP-N-acetyl-alpha-D-galactosamine:polypeptide N-acetylgalactosaminyltransferase 12 | 222773_s_at | -1.5 | AA554045 |
| TMOD3 | tropomodulin 3 | 223078_s_at | -1.5 | AF177171.1 |
| PRO2047 | PRO2047 protein | 226278_at | -1.5 | AI150224 |
| DNCH1 | dynein, cytoplasmic, heavy polypeptide 1 | 229118_at | -1.5 | W93705 |
| ZNF626 | zinc finger protein 626 | 1552643_at | -1.5 | NM_145297.1 |
| MMEL2 | membrane metallo-endopeptidase-like 2 | 1552930_at | -1.5 | NM_033467.1 |
| NKX2-3 | NK2 transcription factor related, locus 3 (Drosophila) (NKX2-3) | 1553808_a_at | -1.5 | NM_145285.1 |
