# Supplementary material for: Genome-Wide Gene Expression Profile Analyses Identify CTTN as a Potential Prognostic Marker in Esophageal Cancer
Source: PLoS One. 2014 Feb 14;9(2):e88918. doi: 10.1371/journal.pone.0088918 (PMC3925182; doi:10.1371/journal.pone.0088918)
Supplement: Table S3 — List of 148 genes associated with cell metabolism. (DOC) [file pone.0088918.s004.doc]

Table S3. List of 148 genes associated with cell metabolism.

| **Gene symbol** | **Gene name** | **Probe set ID** | **Fold-**  **change** | **GenBank no.** |
| --- | --- | --- | --- | --- |
| PNLIP | pancreatic lipase | 205912_at | 5.1 | NM_000936.1 |
| POLE4 | Polymerase (DNA-directed), epsilon 4 (p12 subunit) | 1565329_at | 5.1 | AY034104.1 |
| LIPF | lipase, gastric | 206334_at | 5 | NM_004190.1 |
| PGC | progastricsin (pepsinogen C) | 1555236_a_at | 5 | BC042578.1 |
| DHRS2 | dehydrogenase/reductase (SDR family) member 2 | 214079_at | 4.8 | AK000345.1 |
| CST1 | cystatin SN | 206224_at | 4.4 | NM_001898.1 |
| CTRB1 | chymotrypsinogen B1 | 205971_s_at | 4.1 | NM_001906.1 |
| PGA3 | pepsinogen 3, group I (pepsinogen A) | 213265_at | 4.1 | AI570199 |
| SPINK1 | serine protease inhibitor, Kazal type 1 | 206239_s_at | 3.4 | NM_003122.1 |
| CA9 | carbonic anhydrase IX | 205199_at | 3.1 | NM_001216.1 |
| LOXL2 | lysyl oxidase-like 2 | 202997_s_at | 3 | BE251211 |
| PTPRR | protein tyrosine phosphatase, receptor type, R | 210675_s_at | 3 | U77917.1 |
| TREX2 | clone T2P4 3-5 exonuclease | 211788_s_at | 3 | AF319573.1 |
| CYP26A1 | cytochrome P450, subfamily XXVIA, polypeptide1 | 206424_at | 2.9 | NM_000783.1 |
| HEP27 | Short-chain alcohol dehydrogenase family member | 206463_s_at | 2.9 | NM_005794.1 |
| MME | membrane metallo-endopeptidase | 203434_s_at | 2.8 | AI433463 |
| GAD1 | glutamate decarboxylase 1 (brain, 67kD) | 206670_s_at | 2.8 | NM_013445.1 |
| KIAA1622 | serine/threonine-protein phosphatase 4 regulatory subunit 4 | 220673_s_at | 2.8 | NM_020958.1 |
| TIP30 | Tat interactive protein (30KD) | 229102_at | 2.8 | BG153401 |
| CHI3L1 | chitinase 3-like 1 | 209396_s_at | 2.6 | M_80927.1 |
| TNNI3K | putative protein-tyrosine kinase | 220415_at | 2.4 | NM_015978.1 |
| UCHL1 | ubiquitin carboxyl-terminal esterase L1 (ubiquitin thiolesterase) | 201387_s_at | 2.2 | NM_004181.1 |
| LEPREL2 | leprecan-like 2 | 204854_at | 2.2 | NM_014262.1 |
| CYP27C1 | cytochrome P450, family 27, subfamily C, polypeptide 1 | 1568868_at | 2.2 | BC039307.1 |
| SULF1 | sulfatase 1 | 212354_at | 2.1 | BE500977 |
| NOX4 | NADPH oxidase 4 | 219773_at | 2.1 | NM_016931.1 |
| PRSS21 | protease, serine, 21 (testisin) | 220051_at | 2.1 | NM_006799.1 |
| CST2 | cystatin SA | 208555_x_at | 2 | NM_001322.1 |
| OASL | 2-5oligoadenylate synthetase-related protein p30 | 210797_s_at | 2 | AF063612.1 |
| OLR1 | oxidized low density lipoprotein (lectin-like) receptor 1 | 210004_at | 2 | AF035776.1 |
| GDA | guanine deaminase | 224209_s_at | 2 | AF019638.1 |
| CYP27B1 | cytochrome P450, subfamily XXVIIB | 205676_at | 1.9 | NM_000785.1 |
| SERPINB7 | serine (or cysteine) proteinase inhibitor, cladeB (ovalbumin), member 7 | 206421_s_at | 1.9 | NM_003784.1 |
| PXDN | Melanoma associated gene peroxidasin homolog | 212013_at | 1.9 | D86983.1 |
| NEFL | neurofilament, light polypeptide (68kD) | 221805_at | 1.8 | AL537457 |
| ACP1 | acid phosphatase 1, soluble | 201629_s_at | 1.7 | BE872974 |
| APOC1 | apolipoprotein C-I precursor | 204416_x_at | 1.7 | NM_001645.2 |
| DUSP9 | dual specificity phosphatase | 205777_at | 1.7 | NM_001395.1 |
| TDO2 | tryptophan 2,3-dioxygenase | 205943_at | 1.7 | NM_005651.1 |
| UGT8 | UDP glycosyltransferase 8 (UDP-galactoseceramide galactosyltransferase) | 208358_s_at | 1.7 | NM_003360.1 |
| PON3 | paraoxonase-3 | 213695_at | 1.7 | L48516.1 |
| NY-BR-81 | protein phosphatase 1, regulatory (inhibitor) subunit 14C | 226907_at | 1.7 | N32557 |
| PNKD | paroxysmal nonkinesigenic dyskinesia | 233177_s_at | 1.7 | AB033010.1 |
| CPB1 | pancreatic carboxypeptidase B1 precursor | 205509_at | 1.6 | NM_001871.1 |
| USP18 | ubiquitin specific protease 18 | 219211_at | 1.6 | NM_017414.1 |
| [NUDT11](http://www.ncbi.nlm.nih.gov/gene/55190) | nudix (diphosphate linked moiety X)-type motif 11 | 219855_at | 1.6 | NM_018159.1 |
| OS4 | conserved gene amplified in osteosarcoma | 227609_at | 1.6 | AA633203 |
| MTHFD1L | methylenetetrahydrofolate dehydrogenase (NADP+ dependent) 1-like | 231094_s_at | 1.6 | AL035086 |
| ATP6V0D2 | vacuolar H+ ATPase d2 subunit | 1553151_at | 1.6 | AY079172.1 |
| PIP5K2B | phosphatidylinositol-4-phosphate 5-kinase, typeII, beta | 201081_s_at | 1.5 | NM_003559.1 |
| HK2 | hexokinase 2 | 202934_at | 1.5 | AI761561 |
| APOL1 | apolipoprotein L-I | 209546_s_at | 1.5 | AF323540.1 |
| CTSU | cathepsin U | 210074_at | 1.5 | AF070448.1 |
| APOE | apolipoprotein E | 212883_at | 1.5 | AI358867 |
| BCAT1 | branched chain aminotransferase 1, cytosolic | 214390_s_at | 1.5 | AI652662 |
| OAS3 | 2-5oligoadenylate synthetase 3 | 218400_at | 1.5 | NM_006187.1 |
| GYS2 | glycogen synthase 2 (liver) | 214621_at | -6.2 | S70004.1 |
| TMPRSS11B | transmembrane protease, serine 11B | 1560712_at | -5.2 | AL833167.1 |
| B3GALT2 | UDP-Gal:betaGlcNAc beta 1,3-galactosyltransferase, polypeptide 2 | 210121_at | -4.8 | AF288390.1 |
| SPINK5 | serine protease inhibitor, Kazal type, 5 | 205185_at | -4 | NM_006846.1 |
| PRSS27 | protease, serine 27 | 232074_at | -3.9 | AW170323 |
| ADH1B | alcohol dehydrogenase IB (class I), beta polypeptide | 209612_s_at | -3.7 | M24317.1 |
| EPM2A | epilepsy, progressive myoclonus type 2A, Lafora disease (laforin) | 216079_at | -3.6 | AK022721.1 |
| ADH1A | alcohol dehydrogenase 1A (class I), alpha polypeptide | 209614_at | -3.5 | AF153821.1 |
| PLGLB2 | Plasminogen -like B2 | 1558603_at | -3.5 | AV688060 |
| TKTL1 | transketolase-like 1 | 214183_s_at | -3.4 | X91817.1 |
| TGM3 | transglutaminase 3 | 206004_at | -3.3 | NM_003245.1 |
| SPINK7 | serine peptidase inhibitor, Kazal type 7 | 223720_at | -3.3 | AF268198.1 |
| LOC51190 | neutral sphingomyelinase | 221405_at | -3.2 | NM_016317.1 |
| PPP1R3C | protein phosphatase 1, regulatory (inhibitor) subunit 3C | 204284_at | -3 | N26005 |
| TREH | trehalase (brush-border membrane glycoprotein) | 207378_at | -3 | NM_007180.1 |
| CYP4B1 | cytochrome P450, family 4, subfamily B, polypeptide 1 | 210096_at | -3 | J02871.1 |
| TRHDE | thyrotropin-releasing hormone degrading ectoenzyme | 219937_at | -3 | NM_013381.1 |
| DCT | dopachrome tautomerase (dopachrome delta-isomerase, tyrosine-related protein 2) | 205337_at | -2.9 | AL139318 |
| FCT3A | alpha-1,3 fucosyltransferase 6 | 210398_x_at | -2.9 | M98825.1 |
| TMPRSS2 | transmembrane protease, serine 2 | 205102_at | -2.8 | NM_005656.1 |
| HPGD | hydroxyprostaglandin dehydrogenase 15-(NAD) | 211549_s_at | -2.8 | U63296.1 |
| FMO2 | flavin containing monooxygenase 2 (non-functional) | 211726_s_at | -2.8 | BC005894.1 |
| DESC1 TMPRSS11E | DESC1 protein transmembrane protease, serine 11E | 220431_at | -2.8 | NM_014058.1 |
| CYP11A | cytochrome P450, subfamily XIA (cholesterol side chain cleavage) | 204309_at | -2.7 | NM_000781.1 |
| ALOX12 | arachidonate 12-lipoxygenase | 207206_s_at | -2.6 | NM_000697.1 |
| PDK4 | pyruvate dehydrogenase kinase, isozyme 4 | 225207_at | -2.5 | AV707102 |
| ASPA | aspartoacylase (aminoacylase 2, Canavan disease) | 206030_at | -2.4 | NM_000049.1 |
| PTGDS | prostaglandin D2 synthase 21kDa (brain) | 211663_x_at | -2.4 | M61900.1 |
| LCN1 | lipocalin 1 (protein migrating faster than albumin, tear prealbumin) | 207930_at | -2.3 | NM_002297.1 |
| FUT6 | alpha (1,3) fucosyltransferase | 211885_x_at | -2.3 | U27332.1 |
| SERPINB13 | serpin peptidase inhibitor, clade B (ovalbumin), member 13 | 216258_s_at | -2.3 | BE148534 |
| HMGCS2 | 3-hydroxy-3-methylglutaryl-Coenzyme A synthase 2 (mitochondrial) | 204607_at | -2.2 | NM_005518.1 |
| SERPINB2 | serine (or cysteine) proteinase inhibitor, clade B (ovalbumin), member 2 | 204614_at | -2.2 | NM_002575.1 |
| BBOX1 | butyrobetaine (gamma), 2-oxoglutarate dioxygenase (gamma-butyrobetaine hydroxylase) 1 | 205363_at | -2.2 | NM_003986.1 |
| EPHX2 | clone 129-13 soluble epoxide hydrolase | 209368_at | -2.2 | AF233336.1 |
| CYP2E1 | cytochrome P450-2E1 | 209975_at | -2.2 | AF182276.1 |
| VLCS-H1 | solute carrier family 27 (fatty acid transporter), member 6 | 219932_at | -2.2 | NM_014031.1 |
| ATP6V1C2 | ATPase, H+ transporting, lysosomal 42kDa, V1 subunit C isoform 2 | 1552532_a_at | -2.2 | NM_144583.1 |
| ATP1A2 | ATPase, Na+K+ transporting, alpha 2 (+) polypeptide | 203296_s_at | -2.1 | NM_000702.1 |
| PHYHIP | phytanoyl-CoA 2-hydroxylase interacting protein | 205325_at | -2.1 | NM_014759.1 |
| DNASE1L3 | deoxyribonuclease I-like 3 | 205554_s_at | -2.1 | NM_004944.1 |
| TGM1 | transglutaminase 1 (K polypeptide epidermal type I, protein-glutamine-gamma-glutamyltransferase) | 206008_at | -2.1 | NM_000359.1 |
| CH25H | cholesterol 25-hydroxylase | 206932_at | -2.1 | NM_003956.1 |
| PRSS3 | protease, serine, 3 (trypsin 3) | 207463_x_at | -2.1 | NM_002771.1 |
| CYP2C18 | cytochrome P450, subfamily IIC (mephenytoin 4-hydroxylase), polypeptide 18 | 208126_s_at | -2.1 | NM_000772.1 |
| PRSS4 | protease, serine, 4 | 213421_x_at | -2.1 | AW007273 |
| PLCB4 | phospholipase C, beta 4 | 203895_at | -2 | AL535113 |
| SULT2B1 | sulfotransferase family, cytosolic, 2B, member 1 | 205759_s_at | -2 | NM_004605.1 |
| PYR3 | ryanodine receptor 3 | 206306_at | -2 | NM_001036.1 |
| DIO2 | deiodinase, iodothyronine, type II | 211215_x_at | -2 | AB041843.1 |
| DUOX1 | dual oxidase 1 | 219597_s_at | -2 | NM_017434.1 |
| CYP2C9 | cytochrome P450, subfamily IIC (mephenytoin 4-hydroxylase), polypeptide 9 | 220017_x_at | -2 | NM_000771.2 |
| ST6GALNAC1 | ST6(alpha-N-acetyl-neuraminyl-2,3-beta-galactosyl-1,3)-N-acetylgalactosaminide alpha-2,6-sialyltransferase 1 | 227725_at | -2 | Y11339.2 |
| CYPIIE1 | cytochrome P450IIE1 (ethanol-inducible) gene | 1431_at | -1.9 | J02843 |
| ACPP | acid phosphatase, prostate (ACPP) | 204393_s_at | -1.9 | NM_001099.2 |
| KLK13 | kallikrein 13 | 205783_at | -1.9 | NM_015596.1 |
| SERPINB1 | serine (or cysteine) proteinase inhibitor, cladeB (ovalbumin), member 1 | 212268_at | -1.9 | NM_030666.1 |
| GPX3 | glutathione peroxidase 3 (plasma) | 214091_s_at | -1.9 | AW149846 |
| PPP1R1A | protein phosphatase 1, regulatory (inhibitor)subunit 1A | 205478_at | -1.8 | NM_006741.1 |
| CYP3A5 | cytochrome P450, subfamily IIIA, polypeptide 5 | 205765_at | -1.8 | NM_000777.1 |
| LPIN1 | lipin 1 | 212274_at | -1.8 | AV705559 |
| CLU | clusterin | 222043_at | -1.8 | AI982754 |
| SERPINB11 | serine (or cysteine) proteinase inhibitor, clade B (ovalbumin), member 11 | 1552463_at | -1.8 | NM_080475.1 |
| ALOX15B | 15-lipoxygenase 2 splice variant b | 1555416_a_at | -1.8 | AF468053.1 |
| PCAF | p300CBP-associated factor | 203845_at | -1.7 | AV727449 |
| RNASE4 | ribonuclease, RNase A family, 4 | 205158_at | -1.7 | NM_002937.1 |
| CTSG | cathepsin G | 205653_at | -1.7 | NM_001911.1 |
| MGC10848 | inter-alpha (globulin) inhibitor H5 | 219064_at | -1.7 | NM_030569.1 |
| FLJ20701 | phosphotyrosine interaction domain containing 1 | 219093_at | -1.7 | NM_017933.1 |
| KIAA1307 | ubiquitin protein ligase E3 component n-recognin 4 | 231889_at | -1.7 | AB037728.1 |
| FLJ25179 | alpha-2-macroglobulin-like 1 | 1564307_a_at | -1.7 | AL832750.1 |
| RECK, ST15 | RECK protein precursor | 205407_at | -1.6 | NM_021111.1 |
| HS3ST1 | heparan sulfate D-glucosaminyl3-O- sulfotransferase 1 precursor | 205466_s_at | -1.6 | NM_005114.1 |
| DUSP5 | protein tyrosine phosphatase | 209457_at | -1.6 | U16996.1 |
| ECHDC2 | enoyl CoA hydratase domain containing 2 | 218552_at | -1.6 | NM_018281.1 |
| WFDC1 | WAP four-disulfide core domain 1 (WFDC1) | 219478_at | -1.6 | NM_021197.1 |
| KLK12 | kallikrein 12 | 220782_x_at | -1.6 | NM_019598.1 |
| RDH | retinol dehydrogenase homolog isoform-1 (RDH) | 223952_x_at | -1.6 | AF240698.1 |
| CP | ceruloplasmin (ferroxidase) | 204846_at | -1.5 | NM_000096.1 |
| FABP7 | fatty acid binding protein 7, brain | 205030_at | -1.5 | NM_001446.1 |
| CPA3 | mast cell carboxypeptidase A3 precursor | 205624_at | -1.5 | NM_001870.1 |
| CP | ceruloplasmin (ferroxidase) | 204846_at | -1.5 | NM_000096.1 |
| FABP7 | fatty acid binding protein 7, brain | 205030_at | -1.5 | NM_001446.1 |
| CPA3 | mast cell carboxypeptidase A3 precursor | 205624_at | -1.5 | NM_001870.1 |
| NME5 | non-metastatic cells 5, protein expressed in (nucleoside-diphosphate kinase) | 206197_at | -1.5 | NM_003551.1 |
| CES2 | carboxylesterase precursor | 209668_x_at | -1.5 | D50579.1 |
| ADH7 | alcohol dehydrogenase | 210505_at | -1.5 | U07821.1 |
| FUT2 | Similar to fucosyltransferase 2 (secretor statusincluded) | 210608_s_at | -1.5 | BC001899.1 |
| GPD1L | glycerol-3-phosphate dehydrogenase 1-like | 212510_at | -1.5 | AA135522 |
| GALNT12 | UDP-N-acetyl-alpha-D-galactosamine:polypeptide N-acetylgalactosaminyltransferase 12 | 222773_s_at | -1.5 | AA554045 |
| PRO2047 | PRO2047 protein | 226278_at | -1.5 | AI150224 |
| MMEL2 | membrane metallo-endopeptidase-like 2 | 1552930_at | -1.5 | NM_033467.1 |
