# Supplementary material for: Genome-Wide Gene Expression Profile Analyses Identify CTTN as a Potential Prognostic Marker in Esophageal Cancer
Source: PLoS One. 2014 Feb 14;9(2):e88918. doi: 10.1371/journal.pone.0088918 (PMC3925182; doi:10.1371/journal.pone.0088918)
Supplement: Table S4 — List of 90 genes associated with cell adhesion. (DOC) [file pone.0088918.s005.doc]

Table S4. List of 90 genes associated with cell adhesion.

| **Gene symbol** | **Gene name** | **Probe set ID** | **Fold-**  **change** | **GenBank no.** |
| --- | --- | --- | --- | --- |
| COL10A1 | collagen, type X, alpha 1(Schmid metaphyseal chondrodysplasia) | 217428_s_at | 6.3 | X98568 |
| MMP13 | matrix metalloproteinase 13 (collagenase 3) | 205959_at | 6.2 | NM_002427.2 |
| IBSP | Integrin-binding sialoprotein (bone sialoprotein, bone sialoprotein II) | 236028_at | 6.1 | BE466675 |
| COL11A1 | collagen, type XI, alpha 1 | 37892_at | 6 | J04177 |
| MMP10 | matrix metalloproteinase 10 (stromelysin 2) | 205680_at | 5.3 | NM_002425.1 |
| ITGB6 | integrin, beta 6 | 208084_at | 5.3 | NM_000888.3 |
| MMP3 | matrix metalloproteinase 3 (stromelysin 1, progelatinase) | 205828_at | 4.9 | NM_002422.2 |
| MMP8 | matrix metalloproteinase 8 (neutrophil collagenase) | 207329_at | 4.6 | NM_002424.1 |
| MMP1 | matrix metalloproteinase 1 (interstitial collagenase) | 204475_at | 4.5 | NM_002421.2 |
| CDSN | corneodesmosin | 206193_s_at | 4.4 | NM_001264.1 |
| MMP11 | matrix metallopeptidase 11 (stromelysin 3) | 203876_s_at | 4 | AI761713 |
| ADAM12 | a disintegrin and metalloproteinase domain 12 (meltrin alpha) | 202952_s_at | 3.4 | NM_003474.2 |
| COL1A1 | collagen, type I, alpha 1 | 202310_s_at | 3.3 | K01228.1 |
| MFAP2 | microfibrillar-associated protein 2 | 203417_at | 3.1 | NM_017459.1 |
| LAMC2 | laminin, gamma 2 (nicein (100kD), | 202267_at | 3 | NM_005562.1 |
| nephropontin | nephropontin | 209875_s_at | 3 | M83248.1 |
| OSF-2 | osteoblast specific factor | 1555778_a_at | 3 | AY140646.1 |
| LAMA3 | laminin, alpha 3 | 203726_s_at | 2.9 | NM_000227.1 |
| PLAU | plasminogen activator, urokinase |  |  |  |
| COL5A1 | collagen, type V, alpha 1 | 203325_s_at | 2.6 | AI130969 |
| \COL5A3 | collagen, type V, alpha 3 | 218975_at | 2.6 | NM_015719.1 |
| MMP12 | matrix metalloproteinase 12 (macrophage elastase) | 204580_at | 2.5 | NM_002426.1 |
| SERPINH1 | serine (or cysteine) proteinase inhibitor, clade H (heat shock protein 47), member 1 | 207714_s_at | 2.5 | NM_004353.1 |
| COL5A2 | collagen, type V, alpha 2 | 221730_at | 2.5 | NM_000393.1 |
| FBN2 | fibrillin 2 (congenital contractural arachnodactyly) | 203184_at | 2.4 | NM_001999.2 |
| MMP9 | matrix metalloproteinase 9 (gelatinase B, 92kD gelatinase, 92kD type IV collagenase) | 203936_s_at | 2.4 | NM_004994.1 |
| PLG | plasminogen | 227828_s_at | 2.4 | AV700753 |
| DSC1 | desmocollin 1 | 207324_s_at | 2.2 | NM_004948.2 |
| FAP | Integral membrane serine protease seprase | 209955_s_at | 2.2 | U76833.1 |
| FN1 | fibronectin 1 | 214702_at | 2.2 | AJ276395.1 |
| MMP14 | matrix metallopeptidase 14 (membrane-inserted) | 202827_s_at | 2.1 | AU149305 |
| MATN3 | matrilin 3 | 206091_at | 2.1 | NM_002381.2 |
| SERPINE1 | serpin peptidase inhibitor, clade E (nexin, plasminogen activator inhibitor type 1), member 1 | 202627_s_at | 2 | AL574210 |
| CDH11 | cadherin 11, type 2, OB-cadherin (osteoblast) | 207172_s_at | 2 | NM_001797.1 |
| VCAN | versican | 211571_s_at | 2 | D32039.1 |
| COL1A2 | collagen, type I, alpha 2 | 202404_s_at | 1.9 | NM_000089.1 |
| COL17A1 | collagen, type XVII, alpha 1 | 204636_at | 1.9 | NM_000494.1 |
| BGN | biglycan | 213905_x_at | 1.9 | AA845258 |
| LOX7 | lysyl oxidase (LOX) gene, exon 7 | 215446_s_at | 1.9 | L16895 |
| MGC46341 | discoidin, CUB and LCCL domain containing 1 | 1553768_a_at | 1.9 | NM_173674.1 |
| LAMB3 | laminin S B3 chain | 209270_at | 1.8 | L25541.1 |
| PPFIA1 | LAR-interacting protein 1a | 210236_at | 1.8 | U22815.1 |
| LOC57333 | reticulocalbin 3, EF-hand calcium binding domain | 219102_at | 1.8 | NM_020650.1 |
| DSG2 | desmoglein 2 preproprotein | 1553105_s_at | 1.8 | NM_001943.1 |
| COL4A6 | Similar to collagen, type IV, alpha 6 | 210945_at | 1.7 | BC005305.1 |
| ADAMTS2 | a disintegrin and metalloprotease withthrombospondin motifs-2, isoform 1 | 214454_at | 1.7 | NM_014244.1 |
| COL12A1 | collagen type XII alpha-1 | 231766_s_at | 1.7 | U73778.1 |
| NTF5 | neurotrophin 5 precursor | 231785_at | 1.7 | NM_006179.1 |
| TROAP | trophinin associated protein (tastin) | 1568596_a_at | 1.7 | AI199355 |
| CDH3 | cadherin 3, type 1, P-cadherin (placental) | 203256_at | 1.6 | NM_001793.1 |
| NELL2 | nel (chicken)-like 2 | 203413_at | 1.6 | NM_006159.1 |
| CSPG2 | chondroitin sulfate proteoglycan 2 (versican) | 204619_s_at | 1.6 | BF590263 |
| COL4A1 | collagen, type IV, alpha 1 | 211981_at | 1.6 | NM_001845.1 |
| CLDN1 | claudin 1 | 218182_s_at | 1.6 | NM_021101.1 |
| ITGA11 | integrin alpha-11 subunit precursor | 222899_at | 1.6 | AF109681.1 |
| HXB | hexabrachion (tenascin C, cytotactin) | 201645_at | 1.5 | NM_002160.1 |
| THBS2 | thrombospondin 2 | 203083_at | 1.5 | NM_003247.1 |
| ITGB4 | integrin, beta 4 | 204989_s_at | 1.5 | BF305661 |
| CNTN1 | h-contactin 2 precursor | 211203_s_at | 1.5 | U07820.1 |
| PCDHGC3 | protocadherin 43 | 217426_at | 1.5 | L11372.1 |
| C1ORF10 | chromosome 1 open reading frame 10 | 220090_at | -5.2 | NM_016190.1 |
| MUC5B | mucin 5B, oligomeric mucus/gel-forming | 213432_at | -4.4 | AI697108 |
| ADAM33 | ADAM metallopeptidase domain 33 | 232570_s_at | -3.8 | AL356755 |
| ECM1 | extracellular matrix protein 1 | 209365_s_at | -3.2 | U65932.1 |
| TJP3 | tight junction protein 3 (zona occludens 3) | 213412_at | -3.2 | NM_014428.1 |
| CEACAM1 | carcinoembryonic antigen-related cell adhesion molecule 1 (biliary glycoprotein) | 209498_at | -3 | X16354.1 |
| MMRN | Multimerin | 205612_at | -2.9 | NM_007351.1 |
| CGM2 | carcinoembryonic antigen | 206198_s_at | -2.9 | L31792.1 |
| CEACAM7 | carcinoembryonic antigen-related cell adhesion molecule 7 | 206199_at | -2.9 | NM_006890.1 |
| DPT | dermatopontin | 213068_at | -2.7 | AI146848 |
| MMP27 | matrix metalloprotease 27 | 220783_at | -2.5 | NM_022122.1 |
| TNXB | tenascin XB | 216333_x_at | -2.4 | M25813.1 |
| BGPa | alternatively spliced biliary glycoprotein | 211883_x_at | -2.3 | M76742.1 |
| CLDN10 | claudin 10 | 205328_at | -2.1 | NM_006984.1 |
| ITGA8 | integrin, alpha 8 | 214265_at | -2.1 | AI193623 |
| NLGN1 | neuroligin 1 | 205893_at | -2 | NM_014932.1 |
| CLDN17 | claudin 17 | 221328_at | -2 | NM_012131.1 |
| CEACAM6 | carcinoembryonic antigen-related cell adhesionmolecule 6 (non-specific cross reacting antigen) | 203757_s_at | -1.9 | BC005008.1 |
| MUC1 | mucin 1, transmembrane (MUC1) | 207847_s_at | -1.8 | NM_002456.1 |
| MFAP4 | microfibrillar-associated protein 4 | 212713_at | -1.8 | R72286 |
| ADAMTS1 | similar to Homo sapiens metalloproteinase with thrombospondin type 1 motifs | 222162_s_at | -1.8 | AK023795.1 |
| CLDN5 | transmembrane protein claudin 5 | 204482_at | -1.7 | NM_003277.1 |
| C44A | GPI-anchored metastasis-associated protein homolog | 204952_at | -1.7 | NM_014400.1 |
| NCA | non-specific cross reacting antigen | 211657_at | -1.7 | M18728.1 |
| C4orf31 | chromosome 4 open reading frame 31 | 219747_at | -1.7 | NM_024574.1 |
| XLKD1 | extracellular link domain-containing 1 | 219059_s_at | -1.6 | AL574194 |
| EVA1 | epithelial V-like antigen 1 (EVA1 | 203779_s_at | -1.5 | NM_005797.1 |
| RELN | reelin | 205923_at | -1.5 | NM_005045.1 |
| ITGA7 | integrin alpha-7 | 209663_s_at | -1.5 | AF072132.1 |
| COL14A1 | collagen, type XIV, alpha 1 (undulin) | 212865_s_at | -1.5 | BF449063 |
