# Supplementary material for: Genome-Wide Gene Expression Profile Analyses Identify CTTN as a Potential Prognostic Marker in Esophageal Cancer
Source: PLoS One. 2014 Feb 14;9(2):e88918. doi: 10.1371/journal.pone.0088918 (PMC3925182; doi:10.1371/journal.pone.0088918)
Supplement: Table S5 — List of 64 genes associated with immune response. (DOC) [file pone.0088918.s006.doc]

Table S5. List of 64 genes associated with immune response.

| **Gene symbol** | **Gene name** | **Probe set ID** | **Fold-**  **change** | **GenBank no.** |
| --- | --- | --- | --- | --- |
| CXCL5 | chemokine (C-X-C motif) ligand 5 | 214974_x_at | 4.2 | AK026546.1 |
| IGSF9 | immunoglobulin superfamily, member 9 | 229276_at | 4 | AB037776 |
| SCYB5 | Small inducible cytokine subfamily B, number 5 | 215101_s_at | 3.8 | BG166705 |
| IL11 | interleukin 11 | 206924_at | 3.2 | NM_000641.1 |
| IL8 | interleukin 8 C-terminal variant | 211506_s_at | 2.8 | AF043337.1 |
| TREM2 | triggering receptor expressed on myeloid cells 2 | 219725_at | 2.5 | NM_018965.1 |
| G1P3 | interferon, alpha-inducible protein (clone IFI-6-16) | 204415_at | 2.3 | NM_022873.1 |
| CXCL11 | chemokine (C-X-C motif) ligand 11 | 210163_at | 2.2 | AF030514.1 |
| SCYA3 | small inducible cytokine A3 (homologous to mouse Mip-1a) | 205114_s_at | 2 | NM_002983.1 |
| H174 | putative alpha chemokine | 211122_s_at | 2 | AF002985.1 |
| ZCYTO10，IL20 | four alpha helix cytokine | 224071_at | 2 | AF224266.1 |
| IFIT4 | interferon-induced protein with tetratricopeptide repeats 4 | 204747_at | 1.9 | NM_001549.1 |
| IFIT1 | interferon-induced protein with tetratricopeptide repeats 1 | 203153_at | 1.8 | NM_001548.1 |
| SCYB10 | interferon gamma-induced precursor | 204533_at | 1.8 | NM_001565.1 |
| IL1RL1 | interleukin 1 receptor-like 1 | 207526_s_at | 1.8 | NM_003856.1 |
| PPBP | pro-platelet basic protein (includes platelet basic protein, beta-thromboglobulin | 214146_s_at | 1.8 | R64130 |
| PRV1 | polycythemia rubra vera 1，cell surface receptor | 219669_at | 1.8 | NM_020406.1 |
| SCYA26 | thymic stroma chemokine-1 precursor | 223710_at | 1.8 | AF096296.1 |
| PF4V1 | platelet factor 4 variant 1 | 207815_at | 1.7 | NM_002620.1 |
| AIM2 | absent in melanoma 2 | 206513_at | 1.6 | NM_004833.1 |
| SCYA7 | monocyte chemotactic protein 3 precursor | 208075_s_at | 1.6 | NM_006273.2 |
| DXS1357E | accessory proteins BAP31 | 213843_x_at | 1.5 | AW276522 |
| DEFB3 | beta-defensin 3 | 224239_at | 1.5 | AF301470.1 |
| TNFRSF18 | TNF receptor superfamily activation- inducible protein | 224553_s_at | 1.5 | AF117297.1 |
| MGB1 | mammaglobin 1 | 206378_at | -5.4 | NM_002411.1 |
| TFF3 | trefoil factor 3 (intestinal) | 204623_at | -4.9 | NM_003226.1 |
| SGP28 | specific granule protein (28 kDa); cysteine-rich secretory protein-3 | 207802_at | -4.9 | NM_006061.1 |
| CD163 | CD163 molecule | 216233_at | -4.2 | Z22970.1 |
| CD200R | cell surface glycoprotein receptor CD200 | 1552875_a_at | -4 | NM_138939.1 |
| ARS | ARS component B | 214536_at | -3.8 | NM_020427.1 |
| AZGP1 | alpha-2-glycoprotein 1, zinc-binding | 209309_at | -3.3 | D90427.1 |
| DEFB104 | defensin, beta 104 | 1553521_at | -3.3 | NM_080389.1 |
| KIR2DL5.3 | killer cell Ig-like receptor KIR2DL5.3 | 211410_x_at | -3 | AF217487.1 |
| AK155 | AK155 protein | 221111_at | -2.8 | NM_018402.1 |
| SCYA14 | small inducible cytokine subfamily A (Cys-Cys), member 14 | 205392_s_at | -2.7 | NM_004166.1 |
| FCER1A | Fc fragment of IgE, high affinity I, receptor for; alpha polypeptide | 211734_s_at | -2.7 | BC005912.1 |
| VSIG2 | V-set and immunoglobulin domain containing 2 | 228232_s_at | -2.5 | NM_014312.1 |
| LYNX1 | Ly6/neurotoxin 1 | 1554179_s_at | -2.5 | BC032306.1 |
| IGSF10 | immunoglobulin superfamily, member 10 | 1556579_s_at | -2.5 | AF087980.1 |
| DF | D component of complement (adipsin) | 205382_s_at | -2.4 | NM_001928.1 |
| IL1RN | interleukin 1 receptor antagonist | 212659_s_at | -2.3 | AW083357 |
| KLRB1 | killer cell lectin-like receptor subfamily B, member 1 | 214470_at | -2.2 | NM_002258.1 |
| IL1F6 | interleukin 1 family, member 6 (epsilon) | 221404_at | -2.2 | NM_014440.1 |
| CD1E | CD1e molecule | 215784_at | -2.1 | AA309511 |
| LETAL | lymphocyte effector toxicity activation ligand | 1552777_a_at | -2.1 | NM_139165.1 |
| IL22 | interleukin 22 | 222974_at | -2 | AF279437.1 |
| C7 | complement component 7 (C7) | 202992_at | -1.9 | NM_000587.1 |
| HML2 | macrophage lectin 2 (calcium dependent) | 206682_at | -1.9 | NM_006344.1 |
| CD24 | Cell surface antigen | 209772_s_at | -1.9 | X69397.1 |
| CD1C | CD1C antigen, c polypeptide (CD1C) | 205987_at | -1.8 | NM_001765.1 |
| IL18 | interleukin 18 (interferon-gamma-inducing factor) (IL18) | 206295_at | -1.8 | NM_001562.1 |
| IL8RB | interleukin 8 receptor, beta (IL8RB) | 207008_at | -1.8 | NM_001557.1 |
| DVS27 | DVS27-related protein | 209821_at | -1.8 | AB024518.1 |
| Itln | intelectin | 223597_at | -1.8 | AB036706.1 |
| SCYA19 | beta chemokine Exodus-3 | 210072_at | -1.7 | U88321.1 |
| LANGERIN | Langerhans cell specific c-type lectin | 220428_at | -1.7 | NM_015717.1 |
| KLRC2 | killer cell lectin-like receptor subfamily C,member 2 | 206785_s_at | -1.6 | NM_002260.2 |
| MS4A1 | membrane-spanning 4-domains, subfamily A, member1 | 207496_at | -1.6 | NM_000139.1 |
| HF1 | complement factor H 38-kDa N-terminal fragment | 213800_at | -1.6 | X04697.1 |
| CTRP7 | complement-c1q tumor necrosis factor-related protein (CTRP7) | 223877_at | -1.6 | AF329839.1 |
| CCK1 | CCK1 protein (CCK1) | 224027_at | -1.6 | AF110384.1 |
| PTX3 | pentaxin-related gene, rapidly induced by IL-1 beta (PTX3) | 206157_at | -1.5 | NM_002852.1 |
| DLGAP2 | discs, large (Drosophila) homolog-associated protein 2 | 210227_at | -1.5 | AF119817.1 |
| TCRG | Human T-cell receptor gamma chain VJCI-CII-CIII region mRNA, complete cds. | 209813_x_at | -1.5 | M16768.1 |
